# Inflation of wood resources in European forests: The footprints of a big-bang

**DOI:** 10.1371/journal.pone.0259795

**Published:** 2021-11-24

**Authors:** Jean-Daniel Bontemps

**Affiliations:** IGN, Laboratoire d’Inventaire Forestier, Nancy, France; Kerala University of Fisheries and Ocean Studies, INDIA

## Abstract

The current increase in European forest resources forms a singularity across the globe. Whether this trend will persist, and how biological and economic trends feature it form crucial issues to green economy challenges and C sequestration. The present screening of *Forest Europe* 2015 statistics explored the features, inertia and limits of this expansion, and its relationships with countries’ development, forest management and trade, intense in this area of the world. Persisting footprint of past demographic pressure on forests was identified, with opposed traces on their area and growing stock density. Steady growing stock (GS) increases, proportional to GS, not density-limited, and sustained by forest area increases, supported the view of an inflationary forest dynamic. Economic development and liberalism fostered both forest exploitation and production, yielding no significant impact on GS changes. Wood exports exerted a tension on forest exploitation and GS changes, thus lowering GS inflation but providing a resource security margin in the face of expected climate threats. Conflicting a common view, GS inflation and moderate felling-to-increment ratios make increased use of wood resources and C sequestration reconcilable, and GS expansion timely for ongoing EU forest policy processes. Anticipated adverse impacts of ongoing climate change were not clearly identified in these statistics.

## 1. Introduction

Issues of forest development have been made crucial by the recognition of forests’ provision of socio-economic and environmental services (*Forest Principles* of the Rio conference, [1]). European forests extend over 215 million ha (5.4% of the world’s forests, [2, 3]) with 35 billion m^3^ of accumulated wood (6.5% of global forest growing stock, **GS**), and are therefore denser than the world average. In the European agenda, climate change mitigation [4] and bio-economy [5] strategies are sources of renewed attention to wood production sustainability. They are also matters of debate [6] and recurrent worry among European citizens that prioritize forest preservation and protection objectives [7]. Debates on the new EU forest strategy initiative [8], adopted by the European Commission in 2021, last recall the difficulty to embrace biodiversity, carbon, and circular economy challenges in forest management. Regular forest monitoring as ensured by *Forest Europe* since 1990 and increasingly served by national forest inventory programs (NFI, [9]) has hence turned crucial to European forest policies [10].

As a major outcome of this reporting, both forest area and GS volume have been shown to increase over the recent decades, making forest dynamic across this continent a singularity over the globe ([11], countries with strong forest area increases including China and India show an absence of GS trend, [12, 13]), and incidentally fulfilling the imperative of a “forest greening” [1]. The origin of these changes is allegedly up to centennial, with forest transitions [14] being occurring across most countries since the 19th century [15, 16]. Economic and technologic development and sizable reliance on fossil energies are identified as central factors enabling forest recovery, succeeding long-term population-, agriculture- and industry-driven depletions of forest resources [17]. Also illustrative of this renewal, > 60% of European forests are even-aged and below 80 years in age [18].

These singular ongoing forest changes raise issues of cognitive nature, related to where European forests locate themselves along this long-term expansion process, and how far they situate from both the forest transition turning point, and from any conjectural stationary state depending on future land allocation priorities [19, 20]. Nearly 150 years after early forest transitions, are footprints of ancient pressures exerted on forests still discernible in indicators of forest status or can we clear the past? Also, does current forest dynamic demonstrate early signs of saturation such as a slowing-down, or density-dependent growth limitations?

Well-established from global forest reporting, wood consumption is also stimulated by economic development, with major wood consumers being those among most developed countries [11]. Global concern for the sustainability of forest management in view of intensification strategies [21] and international trade [22] questions this influence of economic development on wood drain from forests, and whether it may slower or even thwart trends in wood accumulation across European forests. Conversely, can signs of an anticipative—or *Boserupian*—forest development ([17], see **Box 1**) be identified that would sustain increases in wood availability? Despite debatable methods, recently reported increases in harvested forest areas in Europe [23] make the question pressing.

**Box 1. Main indicators and concepts of forest dynamic and economic development used in this study. Indicator abbreviation when used, SI unit, description and additional information provided. Only PRIF indicator is external to Forest Europe data 2015**

**Indicators of forest status and dynamic**

**Growing stock (****GS****, m**^**3**^). Standing living wood volume in forests. Terminology used to qualify the ability of living trees to expand in size.

**Growing stock density (GSD, m3.ha**^**-1**^**)**. Spatial density of the growing stock relative to total forest area (international FAO definition). “Forest density” as a terminology also used in the *Forest Identity* metrics (Kauppi et al. 2006). The indicator in 2010 was considered.

**Annual rate of GS change (%.year**^**-1**^**).** Difference in GS at two dates expressed as a fraction of initial GS and annualized. Analysis of rate of GS change 2005-2015 central to this study. “Inflation” is used as a terminology to qualify a strong and continuous increase in GS, mirroring standard uses in economics and cosmology.

**Felling and net increment rates (%.year**^**-1**^**).** Volume of felling and net increment (gross production minor natural tree losses) expressed as a fraction of initial GS and annualized. To comply with GS change definition, indicators in 2010 were considered.

**Felling-to-net-increment ratio (FIR, %.%**^**-1**^ **or unitless).** Conventional forest ratio that measures felling relative to forest production. This indicator should not strongly exceed 100% over the long run. Value of indicator in 2010.

**Acceleration in GS change (%.year**^**-2**^**).** Quantifies the temporal change in the rate of GS change. Computed as a difference of annual rates of GS change between periods 2005-2015 and 1990-2005.

**Indicators of economic development and liberalism in forestry**

**Gross domestic product per capita (GDPc, €.capita**^**-1**^**).** Standard economic indicator of economic welfare that measures the gross value added (GVA) corrected from subsidies (-) and taxes (+). Value of indicator in 2013.

**Property Rights Index in Forestry (PRIF, %)**. Novel indicator of freedom of action in the forestry sector based on property rights assessment, aggregating access, withdrawal, management, exclusion and alienation rights. The indicator is exogenous to *Forest Europe* reporting, and was extracted from Nichiforel et al. (2018, 2020) with value in 2015. It is documented for 30 out of 39 countries under study.

**Contributions of the primary and full forest sector to GVA (%).** Economic sectors as defined by UN/ISIC (International Standard Industrial classification). With respect to the primary forest sector (management and silviculture), the “full” forest sector also encompasses i) the paper and pulp industry, ii) the industrial wood transformation sector. Values of indicators in 2010.

**Annual import and export rates (%).** Annual volumes of wood exports and imports expressed as a fraction of countries’ growing stock. Forest Europe data not distinguishing exports/imports inner/outer Europe. Imports and exports are expressed on a same scale as felling and net increment rates. Values of indicators in 2010.

**Concepts**

**Forest identity.** A conceptual quantitative and 4-dimensional approach to forest description developed by Kauppi et al. (2006) and including forest area, forest volume of growing stock, biomass and carbon as descriptors, and translated from one another using forest density (m3/ha), allometric biomass ratio (ton/m3) and C concentration.

**Malthusian, Boserupian**. Qualifies trends in demographic/economic development/growth that rely on resources distributed across space. *Malthusian* refers to the Thomas Malthus’ view (*An essay on the principle of population*, 1798) that agricultural resource availability constrains human development whose growth was viewed as exponential. *Boserupian* refers to Esther Boserup’ opposed view (*The conditions of agricultural growth*: *the economics of agrarian change under population pressure*, 1965, DOI:10.4324/9781315070360) that human development will on the contrary foster agricultural resource securing through intensification and technological progress. Concepts have been translated into forest resource analysis by Mather et al. (1999).

The emergency of climate change last emphasizes the effective role of forests in sequestrating carbon [24]. *Green economy* strategies advocating resource use intensification are hence recurrently pointed out as conflicting the *in situ* forest C sequestration option [25]. With European forests undergoing areal and stock changes, reconciling these priorities by increasing wood felling while increasing the forest C stock thus arises as an issue.

Since 1990, *Forest Europe*’s regular reporting on forests has been providing an invaluable source of information, with issue 2015 of this report [2] informing >50 indicators over the periods 1990–2015 and 2005–2015, though often fragmental across the ensemble 46 countries. Over the second period, 3/4th of the European forested area was covered by statistical national forest inventory (**NFI**) programs (**S1 Table**, **S1 Fig**) increasing the reliability of statistics dedicated to forest status and dynamic. These statistics however remain less accurate in former socialist countries of Eastern Europe, where NFI programs are recent or inexistant, and reporting data largely stem from forest management plans ([10, 26], see also detailed screening at country level in **S1 Table**). In this investigation of the report, the aims were i) to highlight the salient features of current forest dynamic in Europe and track biological limitations of relevance to the inference of future trends (section 2), ii) to explore the role of economic development and trade on growing stock dynamic, and options for the balance of substitution and C sequestration functions of forests (section 3).

## 2. Insecure past, ongoing inflation and a promising future both footprinted in European forest statistics

### 2.1 Persisting legacies of human population pressure on Europe’s forests

**Fig 1:**
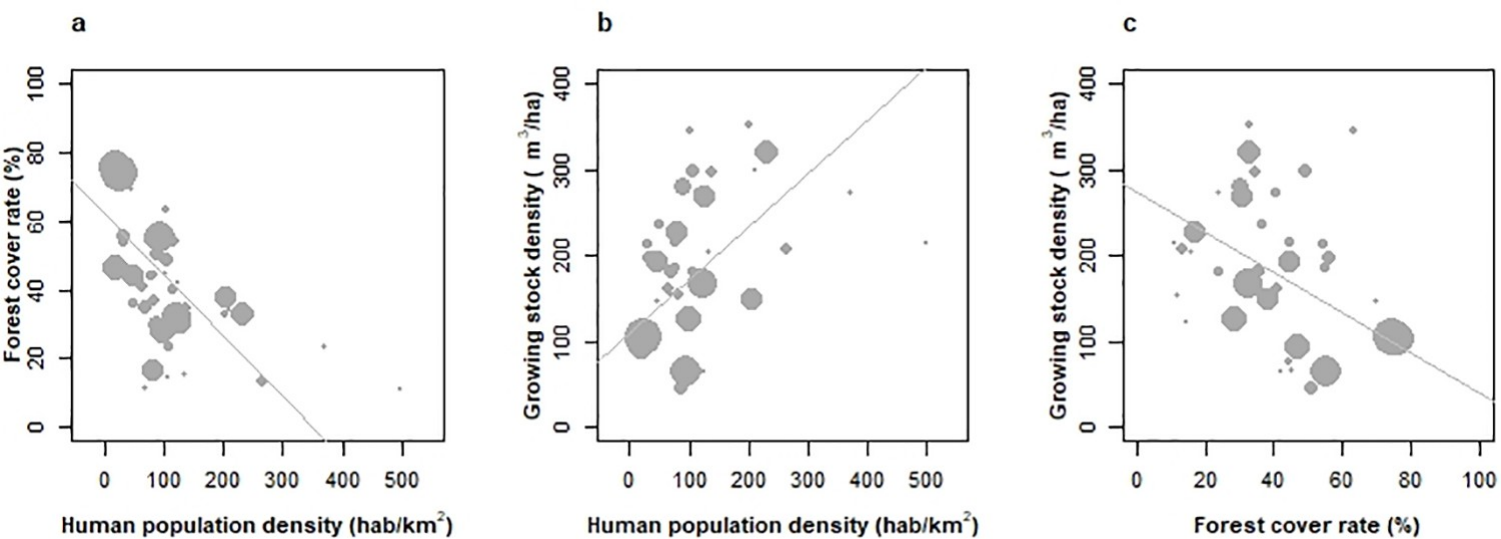
Relationships between human population density and forest area and growing stock across 39 most forested European countries. Correlations weighted by forest area in 2015 are figured. (a) Forest cover **(**fraction of total country area occupied by forests) in 2015 demonstrates a strong negative relationship with population density (weighted correlation -0.64, p < 10^−4^), (b) a strong relationship of opposed sign is found with the growing stock density (weighted correlation +0.54, p < 10^−3^) indicating that growing stock capitalisation has been stronger in countries with smallest forest areas, (c) A trade-off between forest cover and growing stock is accordingly evidenced (weighted correlation -0.56, p < 10^−3^).

A negative relationship was found between population density and the forest cover fraction across countries (**Fig 1A**), attesting a persistent footprint of demographic pressure in the present extension of forests, after decades, sometimes centuries, of reforestation [16]. This relationship was of identical strength with rural population density (correlation –0.64, p < 10^−4^, [27]). By contrast, an unexpected positive relationship was identified between population density and growing stock density (**GSD**, m^3^.ha^-1^, **Box 1**, **Fig 1B,** stronger with rural population density, +0.64, p < 10^−4^) suggesting that the amount of wood resources per unit area was related to population concentration. While a *Malthusian*, or demography-driven forest depletion logic (**Box 1**) has conditioned early trajectories of the European forest area [14, 28], a *Boserupian* logic instead [17] would thus have fostered the increase in wood resources to support nations’ development. The resulting *identity* of European forests [29], **Box 1**) here unmasks an original broad apparent trade-off between the spatial density of forests and their density in wood resources (**Fig 1C**). These effects did not offset each other in countries’ GS, as evidenced by the positive correlation also found between forest area and GS across European countries (+0.69, p < 10^−6^, not shown). Yet, this relationship was found saturating (quadratic) rather than linear (*F*-test of nested models, p < 10^−3^, +9% R2).

### 2.2 The inflationary nature of the European forest growing stock dynamics

**Fig 2:**
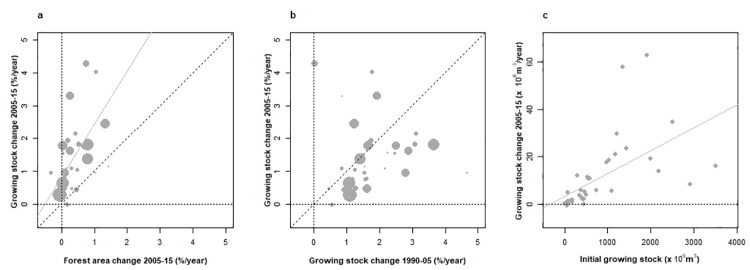
Changes in the growing stock of European countries under study and their comparison with forest area changes and across time. (a) While both the rates of forest area and growing stock changes over 2005–2015 predominantly positive, growing stock change remains greater than that of forest area (slope of regression weighted by forest area in 2015: +1,62 (p < 10^−5^). (b) Rates of change in the growing stock over the two successive periods 1990–2005 and 2005–2015 demonstrate significant inertia (correlation weighted by forest area in 2005: +0.46 (p < 0.01, excluding Romania with growing stock shift generated by NFI implementation). (c) Absolute growing stock change against absolute growing stock shows a proportional trend suggesting capital-driven open dynamic (slope of regression: p < 10^−7^): +0.57 (p < 10^−3^).

Rates of changes in the forest area of European countries over 2005–2015 were found predominantly positive and ranged between –0.3 to +3.2%.year^-1^ (average +0.4%.year^-1^, **Fig 2A**). Though much less popularized (e. g. [13]), rates of GS change over the same period showed a remarkably greater magnitude than those in area with +1.62% GS change.% area change^-1^ (average +1.57%.year^-1^, **Fig 2A**). This ongoing increase in forest area, through the process of tree development, already confirmed a positive component to GS changes in European forests for the future.

Rates of GS change compared across the two reporting periods were found positively correlated (+0.46, p < 0.01, **Fig 2B**) and showed a comparable magnitude (+1.54%.year^-1^ on average over 1990–2005). GS changes therefore demonstrated a substantial inertia, together with a stationary pace.

Last, absolute GS changes over 2005–2015 were found strongly correlated (+0.57, p < 10^−3^) with initial GS (2005, **Fig 2C**), without any clear deviation from a proportional assumption. This suggested that large-scale GS changes remain positively conditioned by GS capital.

Hence European forests exhibited positive GS changes, greater than areal changes, steady over at least 25 years, and positively related to the initial GS capital. Put together, these elements support the view of an open inflationary dynamic which, in essence and in view of its origin (the forest minimum of the forest transition, [14]), portray an apparent forest *big-bang*.

### 2.3 In search of early limitations in GS increases

While GS accumulation in European forests was evidenced (**Fig 2**), causal limitations, either endogenous or exogenous to forests, were also investigated.

**Fig 3:**
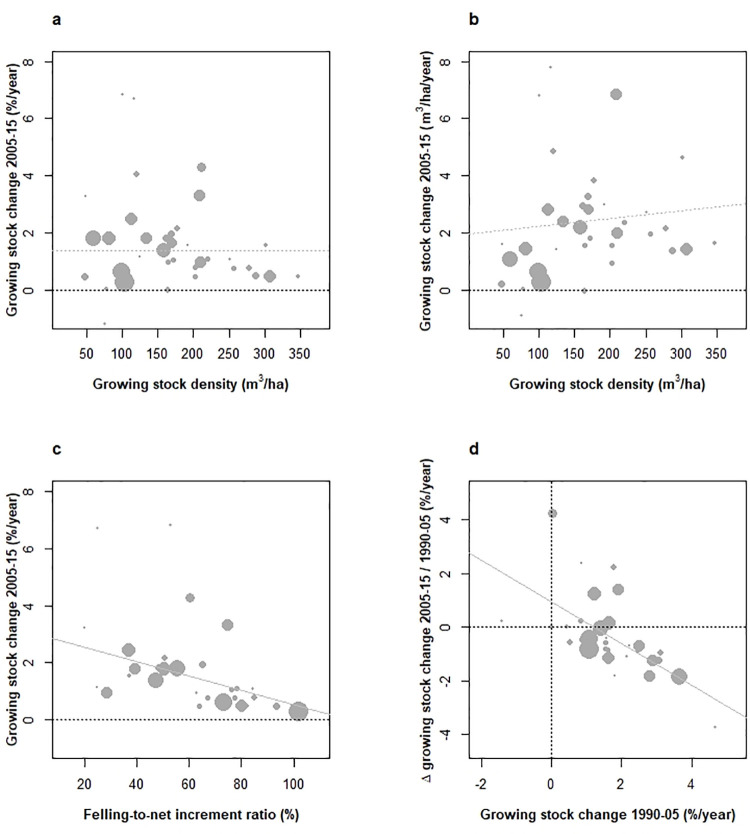
Changes in the forest growing stock of European countries under study over 2005–2015 and their dependence on forest dynamic attributes. Relationships between annual rates of change in the growing stock (2005–2015) against growing stock density (a), felling- to-net-increment ratio (c). Change in the growing stock (2005–2015) was also expressed per hectare (b) for a cross-comparison with (a). Acceleration in growing stock changes over the two successive periods 1990–2005 and 2005–2015 (difference, %.year^–1^) against initial growing stock changes (d). Weighted correlations are: (a) 0.00 (NS), (b) +0.36 (0.02), (c) -0.44 (p < 0.01), (d)– 0.59 (p < 10^−4^). NS correlations figured as dotted lines.

As a biological limitation, growing stock density (**GSD, Box 1**) effect on biomass accumulation was first scrutinized. This relationship also termed *capital-production* or *density-biomass relationship* [30] forms a cornerstone of silviculture [31], whereby biomass production first increases with density, then levels-off and even decreases with exacerbation of competition and mortality [32]. The 95% gradient in GSD was large (50–310 m^3^.ha^-1^, **Fig 3A**) with a modest average 148 m^3^.ha^-1^ across Europe. Only Germany, Switzerland and Austria exceeded 300 m^3^.ha^-1^. Relationships between GSD (2005) and rates of GS change (2005–2015), either expressed per unit of volume (%.year^-1^, **Fig 3A**) or area (m^3^.ha^-1^.year^-1^, **Fig 3B**) showed either null (NS) or even weakly positive (+1,04 m^3^.year^-1^.ha^-1^ per 100 m^3^.ha^-1^ of growing stock, p = 0.02) relationships, respectively. Though lower GS changes in countries of greater GSD were detected, this absence of a density limitation on GS changes was consistent with moderate GSD encountered across most countries, thus not prone to stop GS accumulation in a near future.

Management-driven limitations were inquired using the felling-to-net increment rates ratio (**FIR**, %, 2010, **Box 1**). A large FIR gradient was encountered (**Fig 3C**) and demonstrated a negative association with GS changes (–0.25%.year^-1^ GS change per 10% FIR, p < 0.01), approaching null GS changes with a FIR close to 100%. Of significance, current levels of FIR thus indicated that European forests are able to support increased fellings while maintaining a positive though reduced *in situ* forest GS and therefore C sink. This reconciles overly opposed C substitution/sequestration objectives [33, 34]. With the European strategy for bioeconomy [5] intending to foster use of green renewable resources, current forest dynamic in Europe forms an obvious opportunity in this respect.

In view of the strong and locally impressive pace of GS changes over 2005–2015 (area-weighted average +1.4%.year^-1^, 95% confidence range of 4.6%.year^-1^), we third tracked a possible deceleration in GS changes across periods, using data from period 1990–2005 compared to 2005–2015. Deceleration was measured by the between-period difference of rates of GS change across countries. The mean of this difference was negative (–0.3%.year^-1^) and insignificant (p = 0.8), yet masking a large continental gradient (greatest in Romania/Poland/Turkey with +4.0/+1.4/+1.2%.year^-1^, thus an acceleration, and smallest in Denmark/Ukraine/Spain with –3.7/–1.8/–1.8%.year^-1^). Also, the greater the GS changes over 1990–2005 and the greater the deceleration between periods (**Fig 3D**), whereby acceleration decreased by –0.77% per % annual rate of GS change (R2 = 34%, p < 10^−4^), and was found systematically negative for GS changes beyond +2%.year^-1^. Previous GS change and FIR in 2010 together formed significant predictors of GS acceleration (**Fig 3C**, R2 = 56%), the former being of much greater significance (p = 10^−5^/<0.01, respectively). Possible causes of this apparent deceleration include: i) a negative bias in GS change when switching to statistical inventory methods (see methods and **S1 Fig**), ii) a pattern of *regression to the mean*, after extreme changes in GS over the 1990–2005 period, as often found in observational studies (**Fig 3D**, [35]), iii) climate-related reduction in forest growth [36]), iv) or more dubiously, negative effects of decreases in N deposition [37]. The clear negative asymmetry in this acceleration, and a significant reduction in net volume increment rate in 2010 against that of 2005 (+0.94%.% NI 2005^−1^, p < 10^−10^) were not contradictory to both the method- and CC-based hypotheses. Alltogether, these findings supported the conclusion that GS dynamic in European forests over 2005–2015 has not been statistically preempted by any density, management, or substantial climate limitation.

## 3. Economic development and liberalism generate a controlled tension on wood resources

International reporting on forests [3] has highlighted wood consumption as being highest in most developed economies including Europe, with a likely subsequent pressure on the forest growing stock. However, the continent has also recently shifted from a net importer to exporter of wood [2], suggesting margins in resource availability among countries. These broad features questioned the dependence of GS dynamics on nation’s development and international trade.

### 3.1 Economic wealth association with attributes of GS dynamic

**Fig 4:**
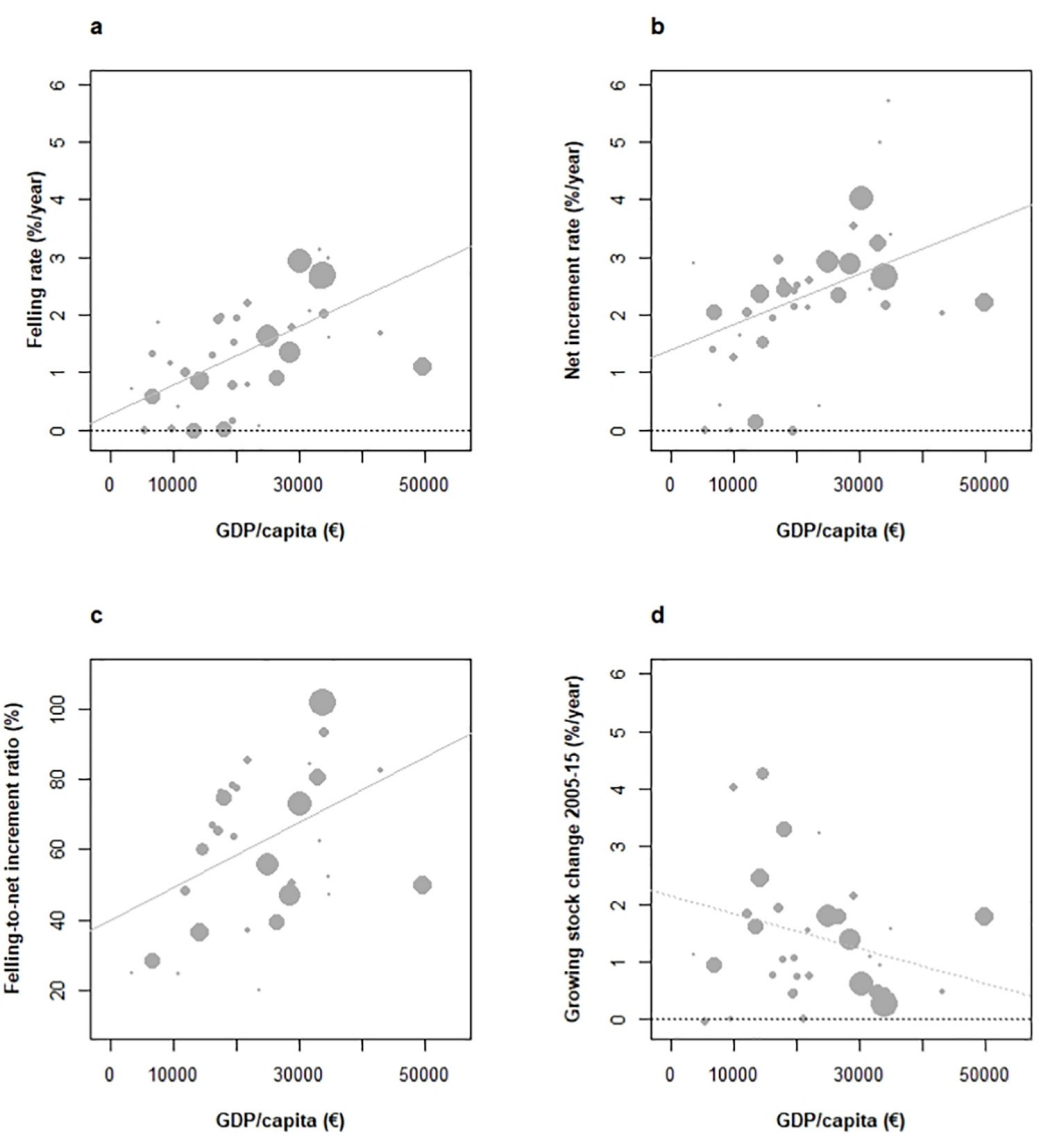
Relationships between GDP per capita and felling and net increment rates (a, b), ratio of felling to net increment (c), and resulting changes in the growing stock over 2005–2015 (d) across European countries under study. Weighted correlations are: (a) +0.57 (p = 10^−4^), (b) + 0.48 (p < 0.01), (c) +0.42 (p <0.05), (c) -0.28 (p < 0.1). NS correlations figured as dotted lines.

Gross domestic product per capita (2013, **GDPc, Box 1**) was first considered in view of its general significance [12]. The dependence of the annual felling rate of forest volume (2010, **Box 1**) on GDPc was found positive and very significant (**Fig 4A**). GDPc did not show any correlation with the extent of forest resources, area or GS, ruling out size effects in this relationship. An order of magnitude in the range GDPc across Europe (95% confidence range between 5.3 k€ and 50.5 k€.GDPc^-1^) hence yielded an increase from +1.3% to +3.1% in the annual felling rate (regression slope +0.051% per 1000€ GDPc^-1^, p < 10^−4^). With GDPc beyond 30 k€.year^-1^, countries including e.g. Sweden, Finland, Germany, or Ireland both showed felling rates above 2.5%.year^-1^, while countries with a GDPc around 15 k€.year^-1^ such as e.g. Turkey, Poland, Ukraine and Romania (>7 Mha forests) showed felling rates below 0.9%.year^-1^. While illegal logging in former Eastern socialist countries may bias felling statistics downward [38], the continued relationship beyond 15,000 € GDPc (high-income countries, **Fig 4A**) suggested greater pressure on forest resources with increasing countries’ richness, even among more advanced economies.

Mirroring this trend, a much less expected positive dependence of the annual net volume increment rate (2010) on GDPc was also identified, though less intense (**Fig 4B**). The same GDPc gradient yielded an increase from +0.25 to +2.2% in the annual net increment rate (regression slope +0.044% per 1000€ GDP.year^-1^.capita^-1^, p < 0.01), and contrasted between Ireland and Denmark (>5%), Finland, Germany (>3%) and Sweden (>2.5%) on the one hand, and Turkey, Romania, Poland or Ukraine (<2.5%) on the other. This again suggested a *Boserupian* trend (**Box 1**) in increasing forest productivity to secure wood resources and meet the economic demand. Both trends however did not counterbalance, and resulted in a weak positive trend in FIR against GDPc (**Fig 4C**, +0.9%.k€ GDPc^-1^, p = 0.02), with countries >30 k€ GDPc such as Sweden, Austria and Germany showing FIR >90%, and countries below 25 k€ in GDPc with large forest areas showing FIR <60% (Turkey, Romania, Spain). Setting Albania aside (FIR of +400% after forest clearance, [39]), FIR ranged between 20% and 100% (101.2% in Sweden). While increasing with economic richness, management intensity thus did not exceed a baseline of 100% FIR across Europe. Accordingly, data showed a negative yet insignificant association between GS changes and GDPc (**Fig 4D**, slope: -0.03%.year^-1^ per k€ GDPc^-1^, p = 0.02), ruling out any broad threat of economic development on wood resources.

### 3.2 A matter of economic richness, or liberalism?

Whereas greater contributions to the gross value added–or GVA–of the primary forestry sector and of total sector (also including wood and paper industry, UN ISIC classification, see **Box 1** for definitions of GVA and forest sectors) were logically found associated with greater forest exploitation (primary sector: + 0.57, p< 0.001, with the felling rate; +0.49, p < 0.01, with the FIR; –0.33, NS with GS changes 2005–2015), these two indicators did not correlate with GDP (–0.005/+0.09, NS), discarding the hypothesis that value added in the forest sector may explain previous relationships of forest intensification with GDPc.

**Fig 5:**
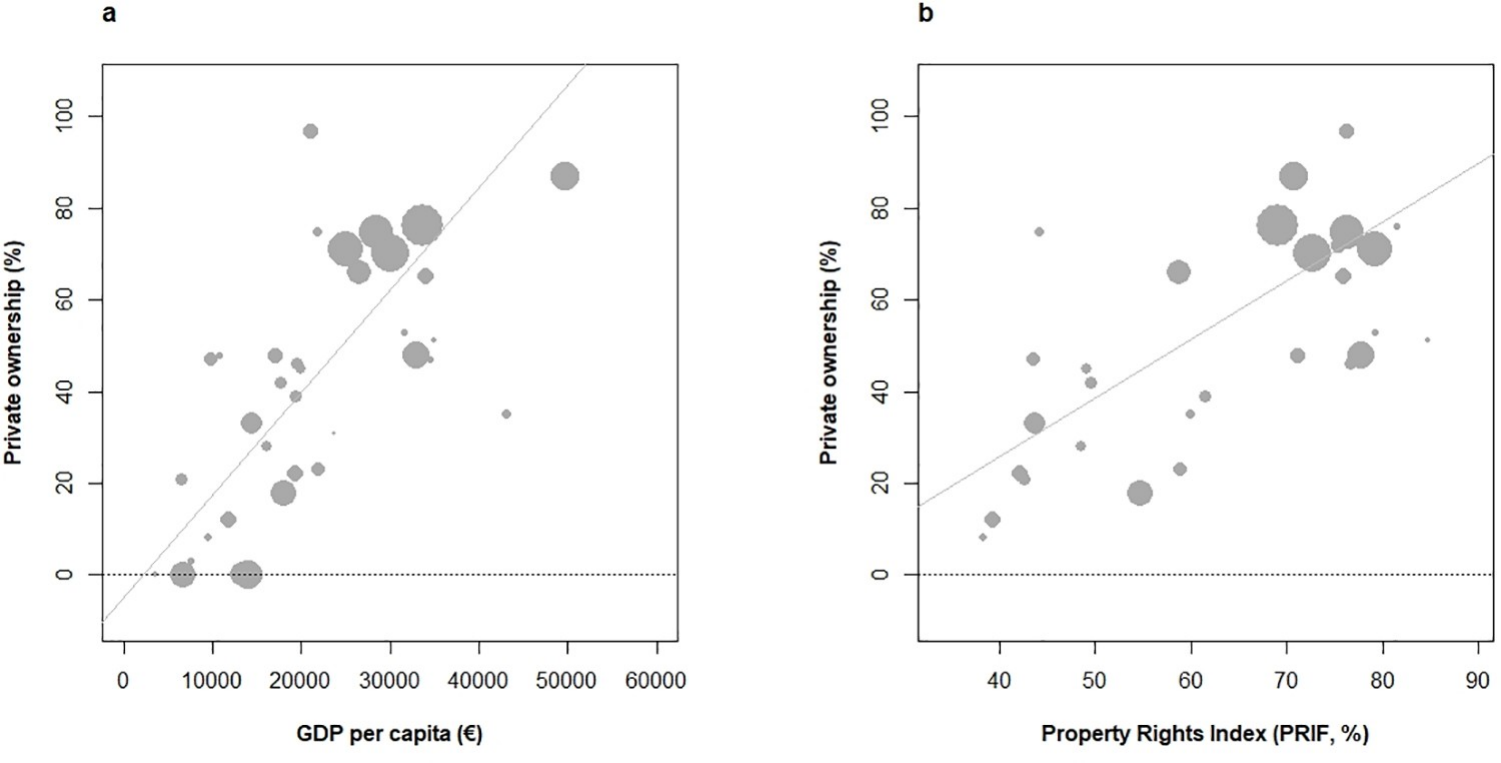
Relationships between private forest ownership fraction and economic richness (a) and freedom in decision making in forestry (b) across European countries under study. (a) economic development as measured by GDP.capita^-1^ (GDPc, euros) in 2013 across 39 countries (b) freedom in decision making as measured by the Property Rights Index in Forestry (PRIF, [42, 43]) in 2015 and available for 30 countries (see methods). Private ownership rate in 2010. Weighted correlations (forest area in 2015): (a) +0.8 (p < 10^−8^) and (b) +0.71 (p < 10^−4^).

In addition, the areal fraction of private forest ownership was found strongly correlated with GDPc (**Fig 5A**, +0.8, p < 10^−8^), to an extent where substituting private ownership fraction to GDPc in previous analyses (**Fig 4**) yielded comparable outcomes (**S2 Fig**). Relationships with FIR/GS rate of change 2005–2015 were again found in limit of significance (+0.36%, p < 0.05)/not significant (–0.24, p = 0.16), respectively.

We therefore hypothesised this association between GDPc and private forest ownership fraction to footprint the existing positive association between economic growth and property rights [40, 41]. Relationships with the novel *property rights index in forestry* (**PRIF**, [42], **Box 1**) as an integrated measure of freedom in decision making across five right categories ([43] for index update in 2015, see methods) were therefore inquired. Property rights remain much more restricted in former Eastern socialist countries to date (with exception of Baltic countries, [42]), yielding a substantial 38–85% gradient across the 30 documented European countries. The mean difference in PRIF between Western (70%) and Eastern countries (45%) thus remained substantial (p < 10^−7^).

Remarkably, PRIF index strongly correlated with the fraction of private forest ownership (+0.71, p < 10^−5^, **Fig 5B**) and to a lower extent with GDPc (+0.59, p < 10^−3^). Again, both the felling and net increment rates were positively associated to this index (**S3 Fig**). Also, GS rate of change 2005–2015 was more negatively associated to PRIF index (-0.37, p = 0.04, **S4 Fig**). The degree of freedom of action–so called liberalism–in forestry was thus found associated to lower GS accumulation in European countries.

In this respect, opposed effects of management plans as a regulation frame were also searched for. While information on forest areal fractions covered by *management plans*/*production management plans* indicators was weak (28/17 countries), these were shown to be positively associated with GSD in 2015 (+0.54/+0.71, p <0.01). FIR/rate of GS change revealed weak yet confirmatory correlations with the areal fraction of production management plans (-0.17, NS; +0.43, p < 0.1, respectively (**S4 Fig)**. Of concern however, a majority of the 17 responding countries were located in Eastern Europe [38], where the absence or novelty of statistical forest inventories programs limit data accuracy (see methods).

As a summary, a trend in greater wood fellings with increasing countries’ richness was evidenced, which was partially offset by greater forest productivity. Thus, GS decrease with increasing countries’ richness–was not significant. GDPc and forest-specific indicators of liberalism (private forest ownership fraction, PRIF) both strongly correlated with each other and produced identical patterns of associations with forest dynamic indicators, making them indistinguishable. FIR further remained in wise limits (< 1) across European countries. Adverse effects of development on GS dynamics were therefore not evidenced. Deciphering the apparent virtuous role of forest management plans on GSD and GS dynamic will require more comprehensive delivery of this indicator across countries.

### 3.3 Growing stock changes in forests further reduced by wood exports

Trade data of *Forest Europe* reporting arise from the *Joint Forest Sector Questionnaire* of the UNECE/FAO protocol [2] and have been subject to debate [44]. These statistics rely on common definitions and are harmonized, making trade indicators the most widely covered among socio-economic indicators across Europe [45].

While wood imports primarily intend to meet inner demands not satisfied by countries’ forests, and in this case without any prior influence on the felling rate or GS dynamic (debatable approach of [44]), they may also support forest protection strategies and unduly amplify GS inflation (e.g. imported deforestation, [46]). Conversely, wood exports may not only arise from production surplus not absorbed by the innner market, but from more offensive trading strategies at a risk of eroding forest GS (e.g. [47]). Crucial sustainability issues are raised in both cases.

**Fig 6:**
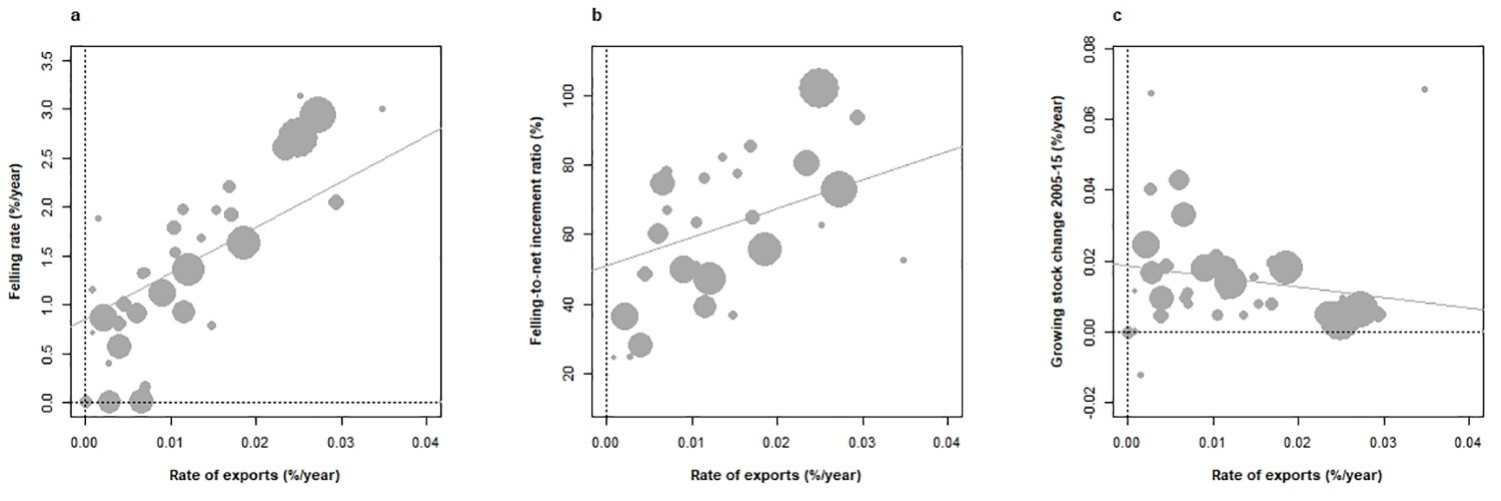
Relationships between the annual rates of wood exports and (a) felling rate, (b) felling-to-net increment ratio and (c) changes in the growing stock over 2005–15 across European countries under study. Both exports, felling and GS changes expressed as fractions (%) of GS volume in 2005. Weighted correlations (forest area in 2015): (a) +0.65 (p < 10–5), (b) + 0.49 (p < 0.01), (c) –0.34 (p < 0.05).

Volumes of imports/exports of wood and derived products expressed as roundwood equivalent fractions of countries’ GS (and termed rates, **Box 1**) were correlated to forest dynamic attributes. Relationships between import and felling rates or GS changes 2005–2015 were found inexistent, ruling out strong protective options among countries. By contrast, a large gradient of export rates (between +1.3% and +6.3% among countries with forests > 1 million ha, 2% on average) generated a remarkable set of correlations (+0.65 with felling rates, p < 10^−5^; +0.49 with FIR, p < 0.01; –0.34 with GS changes 2005–2015, p < 0.01, **Fig 6**) confirming that part of GS inflation in European countries is readily exported. Of note, net increment rates again increased with greater export rates (+0.52, p < 0.001), stressing the responsiveness of the forest sector among countries, in a context of absence of erosion in GS (**Fig 3C**). Export rates were found greatest in traditional forest countries (Sweden, Finland, Germany and Austria; > 2.3%) and whereby intensive forestry is implemented (Portugal, Ireland, Belgium; >3.4%). In this set of countries, GS changes 2005–2015 were significantly lower than on average (+0.28%/+1.57%). That the total annual volume of exports (475 million m^3^) roughly matched the annual increase in GS 2005–15 (422 million m^3^) supports the existence of an ample security margin on wood resource availability in Europe.

## 4. Discussion

### 4.1 The footprint of demographic pressure

The long-lasting pressure of human demography on forests, and its release following industrial/urban development and rural land abandonment, have been early emphasized in studies of forest transitions [14, 27], viewed as an early indicator of demographic transitions [48]. As a later phase of demographic transition, the onset of human fertility fall-down occurred latest in the 1920-1930s in Europe [49]. Forest recovery by up to +50% in area over one century is also well attested in several countries ([50] in Sweden, [51] in Switzerland; [52] in France; [53] in Germany) and is in line with a current pace of +0.5%.year^-1^ in the forest area of most European countries (**Fig 2A**). That a clear negative relationship between forest cover fraction and population density remains apparent in today’s statistics (**Fig 1A**) attests of the irreversible impact of human expansion on forests. In this context, the pressure towards land sparing for nature recovery [54] and the call for spontaneous landscape rewilding in Europe [55, 56], now on the policy agenda (EU-parliament resolution of 2009, [57] are not unsubstantiated. Persisting agricultural land abandonment and returning forests on the European continent [58, 59] form an opportunity in this respect.

In view of this demographic footprint, the positive relationship evidenced between population densities and forest GSD (**Fig 1B**) and the apparent area-GSD trade-off (**Fig 1C**) remain far less expected, and recall the need for more inclusive descriptions of forests (the *forest identity* of [29], **Box 1**). While one cannot exclude that greater densities in forests of the transitional minimum were favoured by historical protection measures, the 200 m^3^.ha^-1^ gradient in GSD observed across countries of the continent suggests that active planning efforts were also implemented to restore timber resources, and within restricted time periods after forest transitions. This is in accordance with Grainger’s [60] distinction between processes reducing forest area and those favouring forest recovery for timber, and with the early enforcement of productive forest management in European forestry laws and practices [15]. Associations of GS attributes with the fraction of countries’ forests under a production management plan also support this view (**S4 Fig**). The predominantly even-aged structure of European forests (70% of area) and its fair distribution across developmental stages (20% regeneration, 60% intermediate, 20% mature forests) lasts suggest a constant effort at renewing forests after final harvest [2, 61].

### 4.2 The origins of GS inflation

#### 4.2.1 A delayed effect of forest transitions

Stock increase causally requests either the addition of new forests with ongoing tree recruitment, or a positive balance between felling and increment of pre-existing forests, resulting from either restricted wood utilization or increase in forest productivity, with possibly endogenic dynamic or environmental causes. While a full quantitative appraisal of each component’s contribution to GS changes remains a scientific challenge [62], the present study already provided some decisive insights.

Consistent with forest transition dating [16], increasing GS in most European countries showed substantial coupling with forest area increase (**Fig 2A**), and was more than double in magnitude. GSD has also greatly increased in Europe over recent decades (148 m^3^/ha in 1990, 171 m^3^/ha in 2005, 192 m^3^/ha in 2015). Such patterns would not be observed early after forest transition, where the contribution of areal extension of forests to GS is minor if any (e. g. transitions in Viet-Nam, China or India and associated area-GS trends in [29]), and should even reduce countries’ forest GSD. The delayed essence of previously afforested areas’ contribution to current GS changes thus has substantial support.

#### 4.2.2 The role of sustainable forest management and physical environmental constraints

With an average FIR of 0.65 in 2010 and below unity in almost all countries (**Figs 3** and **4**), the contribution of restricted current wood felling levels to GS increases makes no doubt, and conflicts recent views [23]. Only in Sweden/Austria, ratios of 101%/93% indicated felling rationalization. Ancient dissemination of sustainable forest management principles certainly features provident felling levels on the continent [63]. Countries reporting the share of forests under production management plans also indicate FIR ratios below 0.8, declining with this share (**S4 Fig**). Average statistics however hide strong within-country heterogeneities [64], including the well-identified and lower intensity of management in forest mountain ranges. Also, forest regrowth on marginal lands abandoned for decades [59] operates on areas physically constrained by terrain ruggedness or soil quality, which hampers forest management intensification [64]. Felling levels in Europe thus appear to result from both active management prescriptions and spontaneous dynamics on isolated areas. This suggests that a fraction of continental forest GS, at least from a resource utilization perspective, remains out of exploitation.

#### 4.2.3 Environmental enhancement of forest growth

Environmental enhancement of the growing stock [65] and more stringently forest growth has been evidenced from major national forest inventories across the continent in dedicated studies embracing temporal periods longer than those covered in standard forest reporting [66–70], i.e. the main data source of *Forest Europe* reporting, with a magnitude of ca. +50% change over 50–100 years. Stimulating roles of nitrogen deposition onto forests [71], atmospheric CO_2_ increase, and climate warming have been put forward as major causes. Both global climate warming and net CO_2_ emissions resulting from ancient deforestation have actually occurred much earlier than usually alleged [72], therefore accompanying forest transitions all along their development. They are also prone to stimulate forest regrowth [73]. Conversely, a more recent null association between GS changes and temperature trends at global scale, even negative across the European continent, was highlighted [12], and may reflect much more recent adverse consequences of climate change onto forest growth, as recently evidenced in Western Europe from national forest inventory studies [74]. Consistently, whereas the net forest volume increment of the countries under study showed a positive difference between 2005 and 2010 (756 resp. 872 million m^3^, p = 0.01) in line with an increasing growing stock, the difference in the net increment rate (**Box 1**) was also found reversed and significant (2.7% in 2005 resp. 2.25% in 2010, p < 0.01), suggesting a decreasing return of the growing stock. More in-depth scrutiny of this decrease and of whether it will last is thus requested.

#### 4.2.4 An unconstrained forest dynamic

Forest dynamics on its own is also able to cause such substantial GS increases, including the increase of forest increment with increasing GS in the absence of density-dependent limitations. This obviously occurs in European forests, as supported by the proportional relationship between GS changes and GS (**Fig 2C**) and the lack of evidence for density-dependent limitations (**Fig 3A** and **3B**), in generally young (predominant age class <80 years, [18]), and not severely stocked forests (most countries’ forests below 200 m^3^/ha, **Fig 3**). The recent analysis of forest dynamic in tall natural forests of New-Zealand [32] forms a precious indication on the upper bounds of GSD in this respect, as it shows that GS equilibrium is reached at an average estimated GSD of 390 m^3^/ha, and in a range of 300–500 m^3^/ha at the extremes (**S3 Table**). These values remain far above the current average GSD in Europe (192 m^3^.ha^-1^ in 2015) where only German, Swiss and Austrian forests exceed 300 m^3^.ha^-1^. GS change inertia relative to the 1990–2005 time period (**Fig 2B**), where no substantial climate change damage onto forests has been reported, also supports a long-term self-sustained dynamic. While not significant, GS deceleration was negatively associated with GS changes over 1990–2005 (**Fig 3D**) and formed a discrete sign, yet not fully elucidated (see 1.3), of a future deflation. Budget-oriented analyses of GS form an appropriate way to quantify endogenous and environmental causes of growth increases. In the single published attempt in this respect to date, Henttonen et al. [75] demonstrated that factors of forest dynamic (growing stock and silviculture) and environmental drivers may have respective contributions of 60/40% to 40-year increases in GS in Finland. Denardou [76] in France supports a contribution of environmental changes of 30%.

#### 4.2.5 The threat of climate change

With increased confidence in the attribution of severe weather events–droughts, heatwaves and storms–to climatic change [77], climatic threats posed on forests and subsequent disturbance regimes (e. g. insect outbreaks) may strongly increase in the future [78], as already evidenced in a recent past in Europe (2003 heatwave in [79], windstorms in Western Europe in [80], insect outbreaks in [81]). The ongoing bark beetle outbreak in Europe and its dependence onto warmth [82] is also illustrative of such consequences. While extending in area and GS capital, European forests also critically show greater exposure to such risks [83]. The magnitude of resulting damages has nevertheless remained very moderate up to 2015, with 0.27%/0.5% of the total forest area affected by fire/storm damages [2], much behind wildlife and grazing pressure (1.4%). Impressive and dramatic though they may be, major storm damages in Europe in the early 21st century (storms *Lothar*, *Martin*, *Gudrun*, *Kyrill*, *Klaus and Cynthia*) amount to 310 million m^3^, i.e. 0.88% of the total GS. A comparable order of magnitude may be reached regarding bark beetle damages in Europe (www.forestry.com/editorial/bark-beetle-invades-europe). With undeniable but not yet substantial climatic impact, and a strong inner dynamic, GS increases may therefore slow down but surely persist in the near decades. The tension exerted by wood exports on countries’ forests (**Fig 6**) attests of a resource security margin for currently exporter countries in the face of future climate threat, albeit export reduction would be prejudicial to international exchanges.

### 4.3 The role of economic development and trade on GS changes

GDPc, the share of private forest ownership, and PRIF as an indicator of freedom of action in forestry were found undistinguishable **(Fig 5**), a global-scale association [84] interpreted as reflecting growth dependence on economic freedom [41]. Recent retrocession of public forests to the private sector in Central and Eastern Europe fits this model [85]. Yet, public expenditure on forests remains greatest in richest economies [84]. Both proxies significantly correlated with increased felling rates and FIR (p < 0.05 for both, **Fig 4, S2** and **S3 Figs**), but non-significantly with decreased GS changes (p < 0.04 with PRIF index). The more causal and direct significance of private forest ownership fraction and PRIF index than GDPc to forest activities however leads us to favour the hypothesis of liberalism influence in the highlighted patterns of forest dynamic. Export rates, not imports, were found associated with attributes of forest dynamic, and positively associated with increased felling rate and decreased growing stock (**Fig 6**), in a very similar way as with previous indicators. While these fluxes were not distinguished between inner/outer Europe in the European reporting, they indicated that part of GS inflation in exporter countries is not capitalized in the forest. The accuracy of trade data has been inquired and has improved over years [86]. Kastner et al. [44] have drawn attention onto several weaknesses including non-inclusion of illegal logging, sensitivity to roundwood conversion factors, and non-inclusion of furniture products, but concluded to their reliability. The comparison of net importer/exporter countries across Europe between 2010 [44] and Forest Europe [2] also confirmed their strong stability. Significantly greater net increments along these gradients stressed the role of forest intensification strategies, with plantations, pine/spruce dominated tree compositions and increased growing stocks mostly conditioning forest harvesting intensity across Europe [64], but also the effect of sustainable silviculture [87]. This coincided with the *Boserupian* perspective on securing resource availability through innovation and technology [88, 89]. However, that GS increases tended to curve down with indicators of economic development and liberalism forms a novelty (**Fig 4, S2** and **S3 Figs**), not highlighted in Kauppi et al. [12]. On the yet narrower EU-27 richness gradient, harvesting intensity was found independent from economic richness [64]. On a global scale, a broad positive association between economic development and GS change has been observed [12], stressing its essential role in forest greening and C sequestration. A closer look at these data nevertheless confirms a flat albeit decreasing GS change response across richest economies (Fig 2B of [12]) in line with our findings (**Fig 4**). The European agenda for more intensive use of renewable resources [5]) may likely foster wood felling, with growing debate on trade-offs with C sequestration [33] and other ecological functions [90], and the new EU forest strategy as a counterbalance (EP 2020). Current forest dynamic is here shown to comply with both felling intensification and *in situ* forest C sequestration persistence strategies (**Fig 3**), suggesting that the issue has a somewhat ideologic dimension. Continued increasing GS also implies greater wood availability at constant felling rates [91]. Yet, an inverse U-shaped future GS response to economic development may not be excluded.

Along with increasing climatic pressure on forests, current statistics–while supporting the inflationary perspective–suggest that the history of the forest transition and environmental Kuznet curves, figuring out impacts of human development on the environment [17, 92] has not been brought to an end. This uncertainty mirrors the scientific debate on the end of universe inflation in cosmology [93]. Fundamental dependence of human societies on natural resources and increasing climatic threats may here form forests’ very “dark matter” and limit future GS accumulation. As this study shows, European forests have yet come with even more green matter up to now, and for a reasonable future.

### 4.4 Conclusions

Increasing forest GS in Europe is multicausal and results from sustained increases in forest area after the forest transitions, from wood felling rates that remain below increment rates (FIR < 1), and from a still moderate capitalization of forests (192 m^3^/ha) that does not biologically limit volume expansion. Despite there exist strong evidences for a stimulating influence of past environmental changes on forest growth, the time horizon of forest reporting prevented its direct scrutiny.

The footprints of a strategic anticipation of forest resource development are various, and form a clear outcome of relationships of forest status/dynamics with human population density, GDP, private ownership fraction and PRIF (property rights) indicators, whereby increased felling rates also come along with increased increment rates. The degree of richness and liberalism is therefore not identified as a threat on forest resources.

Increased exports nevertheless yield significantly lower GS increases. This exported fraction of GS expansion reveals a security margin in the face of the growing climatic threat posed on forests. Over the studied period, a slightly lower net increment rate in 2010 than in 2005, and a GS change deceleration between 1990–2005 and 2005–2015 formed the single and still discrete candidate indices of increasing environmental stress.

Remarkably, all relationships identified were found unambiguous and consistent between each other, and stress the overall remarkable quality of data assembled in *Forest Europe* reporting, in spite of their large assumed heterogeneities across countries and indicators.

## 5 Materials and methods

### 5.1 Country selection

The *Forest Europe* reporting encompasses the 46 countries of Europe. Forest area is strongly variable across these countries, encompassing a gradient of several orders of magnitude. Forests of the Russian federation that extend up to nearly two orders of magnitude the importance of most significant forest resources in Western Europe were discarded from the analysis, in view of the awkward stationarity of official statistics in both the forest area and growing stock (pp 245 and 249 of Forest Europe, 2015), and the intrinsic difficulties of a federal reporting both homogeneous across space and constant over time [94]. Also, to not overweight the influence of small forests, a lower selection threshold was introduced in view of (1) maintaining the gradient of forest area around two orders of magnitude, (2) considering forest areas above 1% of the average country forest area (5,006 million ha). The threshold was therefore fixed at 50,000 ha. Six countries of anecdotal forest area were therefore removed from the analysis, including: Iceland (49,000 ha), Andorra (16,000 ha), Liechtenstein (6,200 ha), Malta (300 ha), Holy See and Monaco (statistical zeroes). Data from the remaining countries were analysed. A synoptic view of forest inventory program implementation across these 39 countries is provided in **S1 Table**.

### 5.2 Influence of statistical forest inventory programs in forest reporting

NFI programs are based on fundamental principles of statistical sampling and inference and constitute the most achieved form of forest resource inventory [95]. After early introduction in the Nordic countries in the 1920s, these programs have disseminated up to recently [9], including Eastern Europe (e.g. Poland, Romania) and countries with restricted forest cover (e.g. in Iceland), and are also at a project stage for others (e.g. Ukraine and Turkey, **S1 Table)**. While NFI programs covered the full reporting period 1990–2015 in only 11 countries under scrutiny, the later period 2005–2015 was covered in 13 additional ones. Consequently, 54%/72% of the European forest area were covered by NFI programs by 1990/2005, respectively (**S1A Fig**). Countries’ forested area (e.g. Sweden, Finland, France) and economic richness (e.g. Norway, Netherlands, Switzerland) appeared to be fundamental determinants of early implementation of NFI programs (**S1B Fig**). The effects of NFI forest coverage (**S1 Table**) on areal and GS rates of changes at the two reporting periods were screened systematically and did not reveal any significant bias. Yet, lower annual rates of change in GS 2005–15 were observed with an NFI program (–0.54%.year^-1^, p = 0.3), more acute with NFI implemented prior to 1990 (–0.83%.year^-1^, p = 0.2). Also areal changes were found smaller in presence of NFI (–0.36%.year^-1^, p = 0.08) as a likely consequence of implementing a forest definition [96]. Owing to these findings and to the restricted NFI cover over 1990–2005, the reporting period 2005–2015 was privileged in this analysis.

### 5.3 Study period

The reporting period 2005–2015 was therefore privileged in view of the restricted forest cover ensured by NFI programs over 1990–2005 (11 countries among those under study, 54% of the forest covered) and the biases highlighted. For some countries, reporting data on forest fluxes could not result from NFI programs alone, as either launched late after 2005 (e.g. UK) or attesting of only one running NFI cycle (e.g. Greece). The period 1990–2005 was however included in the investigation of GS change inertia (**Fig 2B**) and GS change deceleration (**Fig 4D**). Some indicators published in *Forest Europe* 2015 [2] report were specific to one or some reference years, which we selected to fall into the study period 2005–2015. This concerned forest net increment, felling, and felling-to-net-increment ratios, volumes of wood imports and exports, and contributions of the forest and wood industry sectors to gross value added–GVA (all in 2010), and indicators of population density and GDP (2013).

### 5.4 Exogenous data

The country-wise *property rights index in forestry* (PRIF) indicator is a measure of freedom of action in forestry, designed by Nichiforel et al. [42], and updated and released for 33 countries for the reference year 2015 [43]. The index ranges from 0 to 100%, and is an arithmetic mean of 37 indicators ranging from 0 (fully restricted rights) to 100% (no restriction), and covering 5 categories of legal rights (access rights, timber and NWFPs withdrawal rights, management rights (silviculture and planning), exclusion rights (public access and NWFPs) and alienation rights (forest land and timber). The index was not available for 9 out of 39 countries under study, including: Turkey, Ukraine, Belarus, Georgia, Montenegro, Albania, Moldova, Cyprus and Luxembourg.

### 5.5 Analyses

All analyses and figures were performed and drawn with R software version 3.5.1 and packages (R Core Team (2018). R: A language and environment for statistical computing. R Foundation for Statistical Computing, Vienna, Austria. URL https://www.R-project.org/). A preliminary and systematic matrix correlation analysis (Pearson’s correlations and associated test) was performed on all quantitative indicators published in *Forest Europe* 2015 and systematically to fairly delivered by European countries. The correlation matrix (Pearson correlations) is provided in **S2 Table**. This process was intended to ensure inclusiveness of the analysis and detect unanticipated relationships, meaningful to the issue of current forest inflation. Weighted correlations and weighted regression analyses were systematically computed, with countries’ forest area in 2015 as the weighting vector across countries most able to fulfil the requirements of the international UN/FAO forest definition owing to the increased implementation of NFI programs across Europe [97]. Weighted correlations and associated t-tests were computed with *weights* package of R. Weighted regressions and associated t-tests and R2 were computed with the standard *lm* function of R. P-values delivered all along the manuscript refer to these tests. Those associated to regression model comparison (GS change deceleration against FIR 2010 and GS change 1990–2005) result from nested F-tests. Significance was assessed at a 0.01 level. Graphical representations were computed with function *symbols* of R, allowing dot sizing with forest area (radius proportional to square root of countries’ forest area). For some relationships (including **Fig 2C** of the manuscript), weighting was unnecessary as soon as the x-indicator was a measure of countries’ forest extension.

## Supporting information

S1 Fig Status and origins of European forest coverage by statistical national forest inventory (NFI) programs. (a) Fraction of the European forest area covered by a national statistical forest inventory program in 1990 and 2005, (b) influence of countries’ richness and importance of forest area on their existence.
(JPEG)

S2 Fig Relationships between private ownership rate (% forest area) and felling (a) and net increment (b) rates, felling-to-net-increment (FIR, c) and resulting changes in the growing stock over 2005–2015 (d) across 39 European countries under study. Private ownership rate in 2010. Albania was set aside (see Methods) Weighted correlations (forest area in 2015): (a) +0.60 (p < 10^−4^), (b) +0.55 (p < 10^−3^), (c) +0.36 (p < 0.05), -0.24 (p = 0.16).
(JPEG)

S3 Fig Relationships between the Property Rights Index for Forests (PRIF, scale ranging from 0 to 100) and felling and net increment rates (a, b), felling-to-net-increment (FIR, c) and resulting changes in the growing stock over 2005–2015 (d) across a subset of 30 out of 39 European countries under study. PRIF index in 2015, as defined in Nichiforel et al. 2018, and updated for 33 European countries in Nichiforel et al. 2020. Private ownership rate in 2010. PRIF in countries including Turkey, Ukraine, Belarus, Georgia, Montenegro, Albania, Moldova, Cyprus and Luxembourg was not documented. Weighted correlations (forest area in 2015): (a) +0.53 (p < 0.001), (b) +0.69 (p < 10^−4^), (c) +0.04 (NS), -0.37 (p = 0.04).
(JPEG)

S4 Fig Relationship between forest area subjected to a production forest management plan (% forest area) and (a) growing stock per hectare (GSD, m^3^/ha), (b) felling-to-net increment ratio (%), and (c) growing stock change over 2005–2015 for 16 countries reporting on the indicator. FIR in 2010. GSD in 2015. Weighted correlations (forest area 2015) are (a) +0.71 (p < 0.01), (b) -0.17 (NS), (c) +0.43 (< 0.1). 12/16 countries with NFI programs covering the reporting period 2005–2015, 10/16 as EU members, 11/16 located in Eastern Europe: Ukraine, Belarus, Turkey, Bulgaria, Poland, Hungary, Croatia, Slovakia, Slovenia, Latvia and Estonia.
(JPEG)

S1 Table Coverage by statistical national forest inventory (NFI) programmes of the *Forest Europe* reporting periods in the 40 European countries under study. See paper for country selection. ^a^ Data from Forest Europe 2015, ^b^ 1: 1990–2015, 2: 2005–2015, ^c^ C1 = 1^st^ cycle of inventory, C2 = 2^nd^ cycle of inventory. Reporting coverage by NFI statistics assumes that results of a previous NFI cycle were available prior to the starting year of a reporting period. ^d^ Information were obtained from Tomppo et al. 2010 (NFI–pathways for common reporting, Springer) and Vidal et al. 2016 (NFI–Assessment of wood availability and use, Springer) syntheses of European COST actions dedicated to national forest inventories. Links to additional resources are indicated when found.
(DOCX)

S2 Table Correlation matrix of the indicators extracted and computed from *Forest Europe* data 2015. Significant correlations are highlighted in grey (trivial correlations associated to similar proxies or basic size effects, e. g. forest area and growing stock), in red (level of significance of correlation *t*-test <0.01) or green (level of significance <0.05). The test statistics of a Pearson correlation is given by *t* = r^2^/(1-r^2^)^1/2^ (n-2)^1/2^ where r is the correlation and n the number of sampling units (n = 39, see **S1 Table**), yielding: r = *t* / (*t*^2^ + n– 2)^1/2^. Associated correlations thresholds: |r| > 0.32 (p < 0.05) and |r| > 0.41 (p < 0.01). Indicators: A_country = country area (km^2^), EU28/NFI: binary variables indicating EU members and implementation of an NFI program, PopDens/RurPopDens = population density/rural population density (hab/km^2^), GDP = annual gross domestic product (€/capita), %Priv, %MgtPlan, %ProdMgtPlan = share of country forest area private/under a management plan/under a production management plan (%), A = forest area (km^2^), %AFor = country afforestation rate (%), GS = country forest growing stock (m^3^), GSD = country forest growing stock density (m^3^/ha), NI = forest net increment (m^3^), Fell = felling volume (m^3^), FIR = felling-to-net-increment ratio (%), %NI, %Fell = expressed as a percentage of total previous GS, ChgA/ChgGS = absolute changes in A/GS, %ChgA/%ChgGS = changes in A/GS expressed as a percentage of total previous GS, ChgGSha = change in GS expressed per unit of previous forest area. AccGS %/ha = acceleration in GS changes, computed as the difference between annual changes in GS over the two study periods 1990–2005 and 2005–15. Notations _YY/_YYYY refer to the calendar year (state variable) or calendar period (flux).
(DOCX)

S3 Table Conversion of aboveground forest carbon stocks encountered in natural forests of New-Zealand as aboveground forest GS in stem volume. Aboveground forest carbon stocks stem from Paul T., Kimberley M.O., Beets P. N. Natural forests in New Zealand–a large terrestrial carbon pool in a national state of equilibrium. *Forest Ecosystems* (2021): 8:34. These forests are broadleaved with a *Fagus* genus as a dominant tree species, thus a wood specific gravity of 600 kg/m^3^ was used for biomass to volume conversion, using *Fagus sylvatica* data from Kerfriden B. et al., 2021, *Plant Ecology* 222:289–303, DOI: 10.1007/s11258-020-01106-0. The ratio of total aerial tree volume to total stem volume has been estimated at 1.27 in broadleaved semi-natural forests of France including 49 tree species and it was used for stem volume conversion using Saint-André L. et al., 2010, *in* Loustau D. (ed) Forests, carbon cycle and climate change, *Quae* ed, Paris, p79, Table 4.1. Last, conversion of total C stock (including roots, litter and deadwood) to aboveground C stock in Paul et al. arise from the constant 0.6 fraction established from this reference, S3 Table.
(DOCX)
