# Correction: Inflation of wood resources in European forests: The footprints of a big-bang

**DOI:** 10.1371/journal.pone.0307614

**Published:** 2024-07-18

**Authors:** Jean-Daniel Bontemps

The Figs 1–6 are at low resolution. Please see the correct Figs 1–6 here.

**Fig 1:**
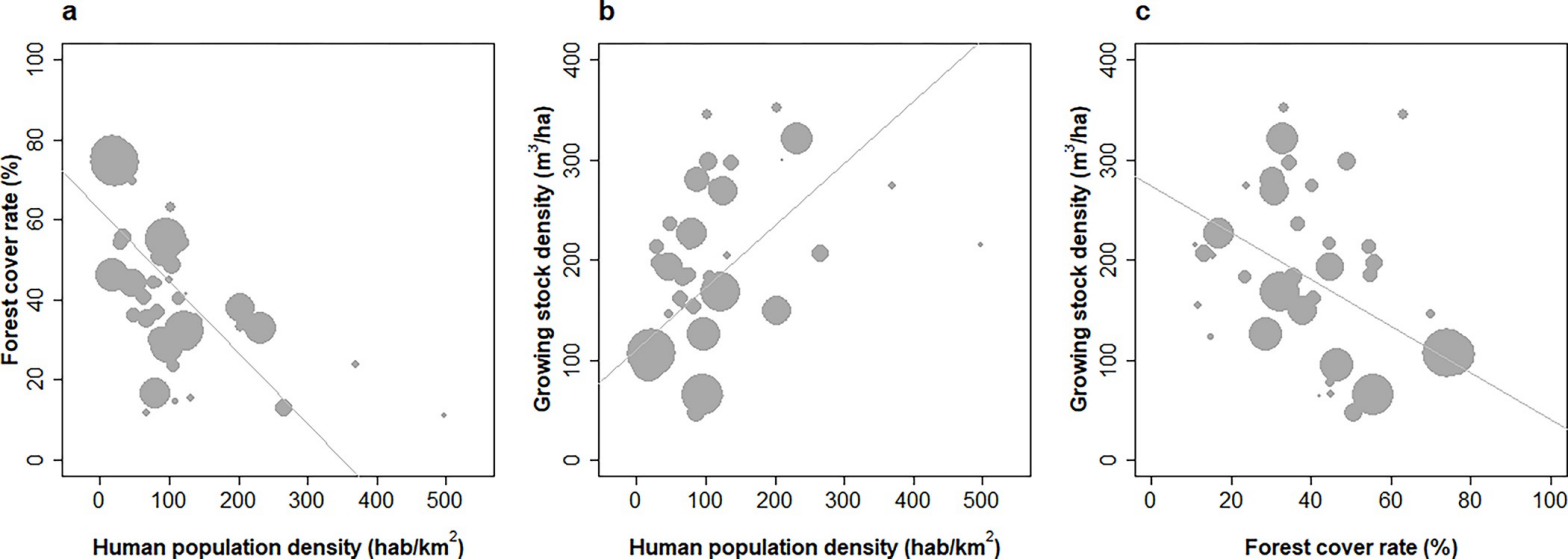
Relationships between human population density and forest area and growing stock across 39 most forested European countries. Correlations weighted by forest area in 2015 are figured. (a) Forest cover (fraction of total country area occupied by forests) in 2015 demonstrates a strong negative relationship with population density (weighted correlation -0.64, p < 10−4), (b) a strong relationship of opposed sign is found with the growing stock density (weighted correlation +0.54, p < 10−3) indicating that growing stock capitalisation has been stronger in countries with smallest forest areas, (c) A trade-off between forest cover and growing stock is accordingly evidenced (weighted correlation -0.56, p < 10−3).

**Fig 2:**
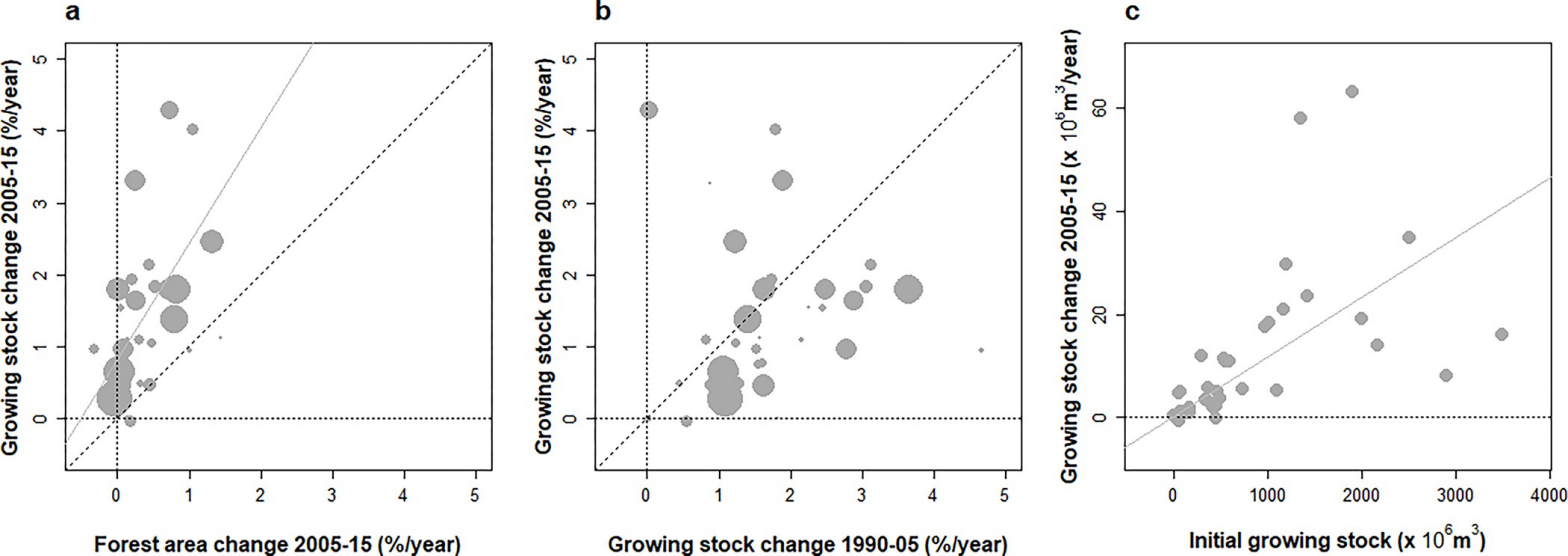
Changes in the growing stock of European countries under study and their comparison with forest area changes and across time.

(a) While both the rates of forest area and growing stock changes over 2005–2015 predominantly positive, growing stock change remains greater than that of forest area (slope of regression weighted by forest area in 2015: +1,62 (p < 10−5). (b) Rates of change in the growing stock over the two successive periods 1990–2005 and 2005–2015 demonstrate significant inertia (correlation weighted by forest area in 2005: +0.46 (p < 0.01, excluding Romania with growing stock shift generated by NFI implementation). (c) Absolute growing stock change against absolute growing stock shows a proportional trend suggesting capital-driven open dynamic (slope of regression: p < 10−7): +0.57 (p < 10−3).

**Fig 3:**
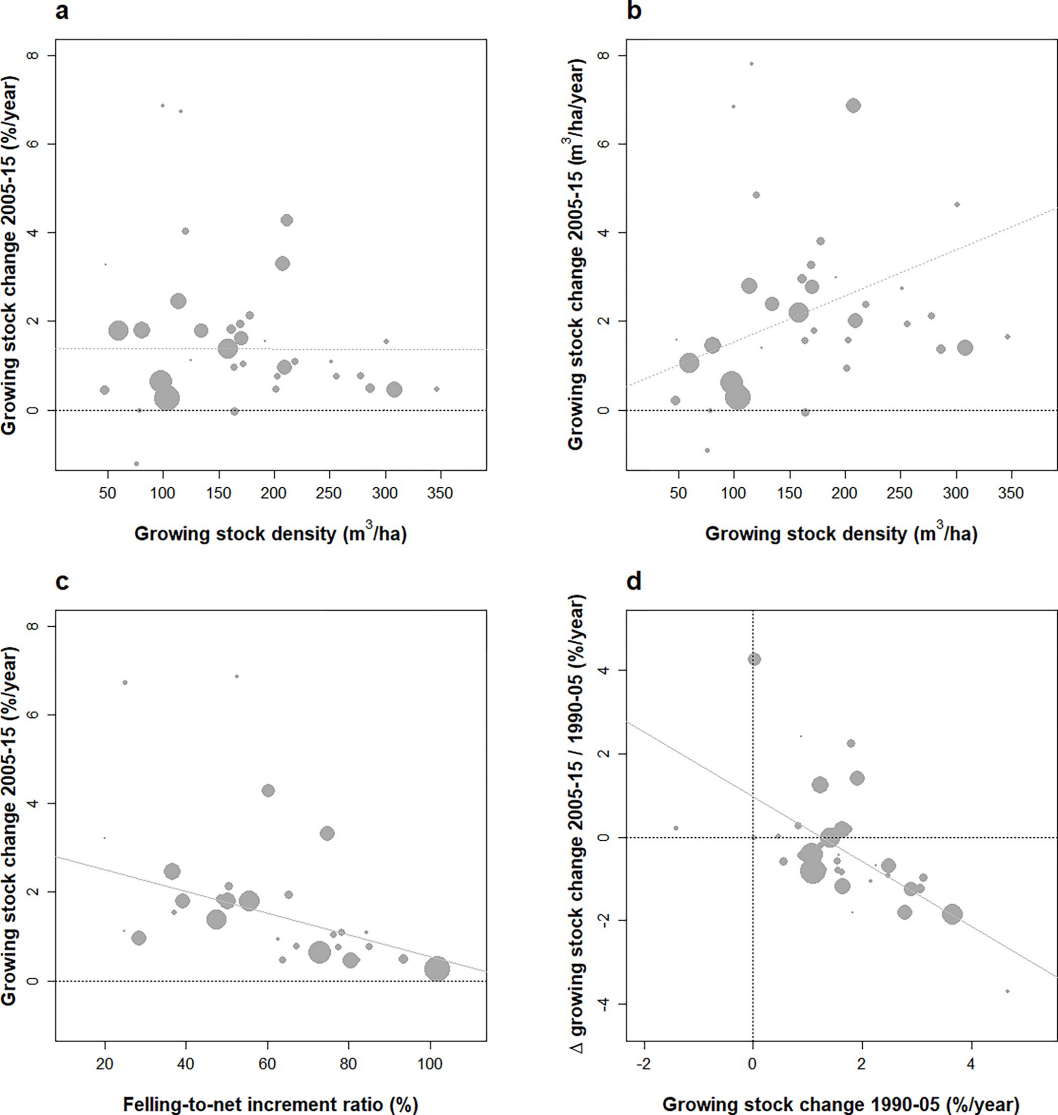
Changes in the forest growing stock of European countries under study over 2005–2015 and their dependence on forest dynamic attributes. Relationships between annual rates of change in the growing stock (2005–2015) against growing stock density (a), felling- to-net-increment ratio (c). Change in the growing stock (2005–2015) was also expressed per hectare (b) for a cross-comparison with (a). Acceleration in growing stock changes over the two successive periods 1990–2005 and 2005–2015 (difference, %.year–1) against initial growing stock changes (d). Weighted correlations are: (a) 0.00 (NS), (b) +0.36 (0.02), (c) -0.44 (p < 0.01), (d)– 0.59 (p < 10−4). NS correlations figured as dotted lines.

**Fig 4:**
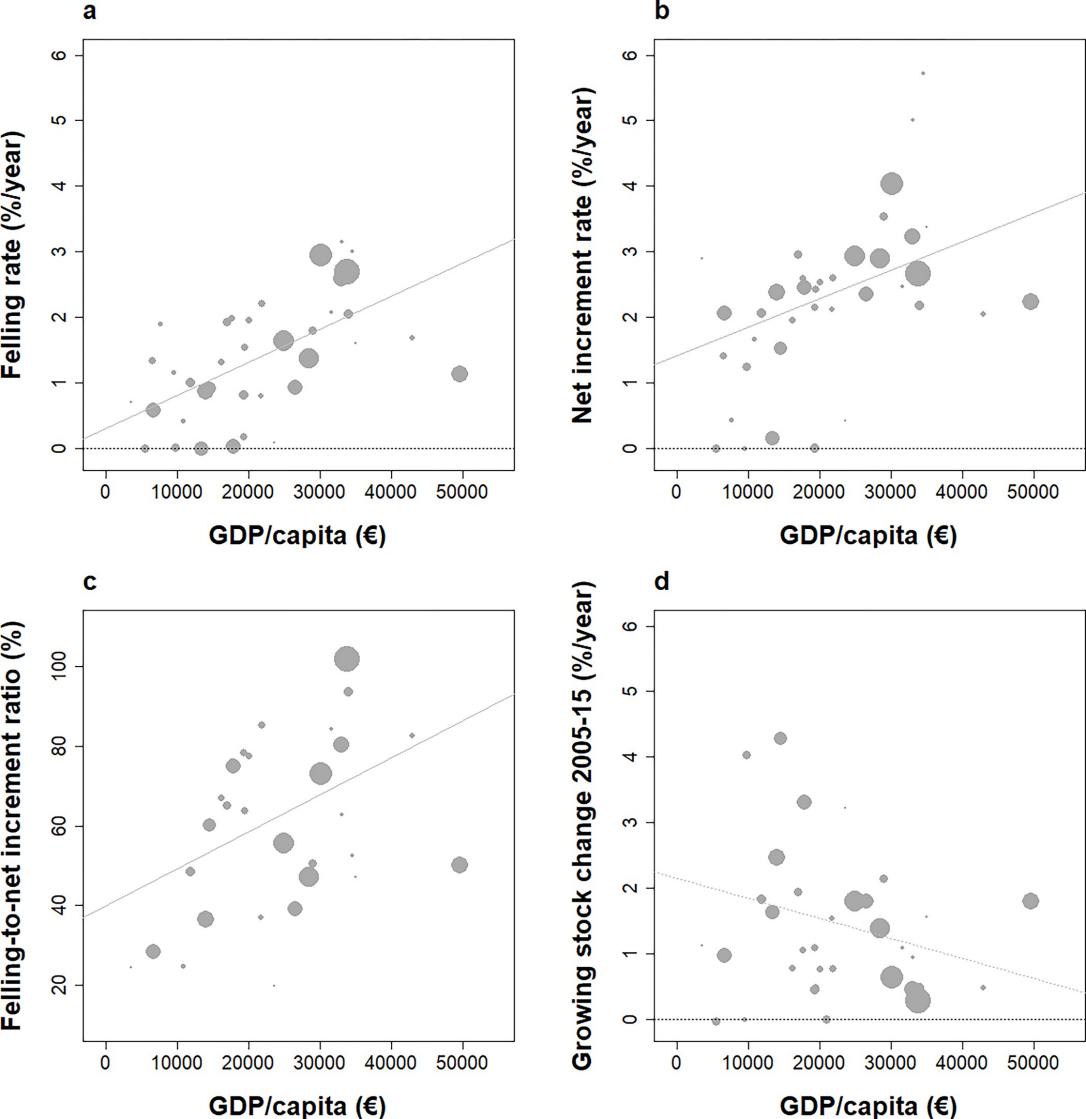
Relationships between GDP per capita and felling and net increment rates (a, b), ratio of felling to net increment (c), and resulting changes in the growing stock over 2005–2015 (d) across European countries under study. Weighted correlations are: (a) +0.57 (p = 10−4), (b) + 0.48 (p < 0.01), (c) +0.42 (p <0.05), (c) -0.28 (p < 0.1). NS correlations figured as dotted lines.

**Fig 5:**
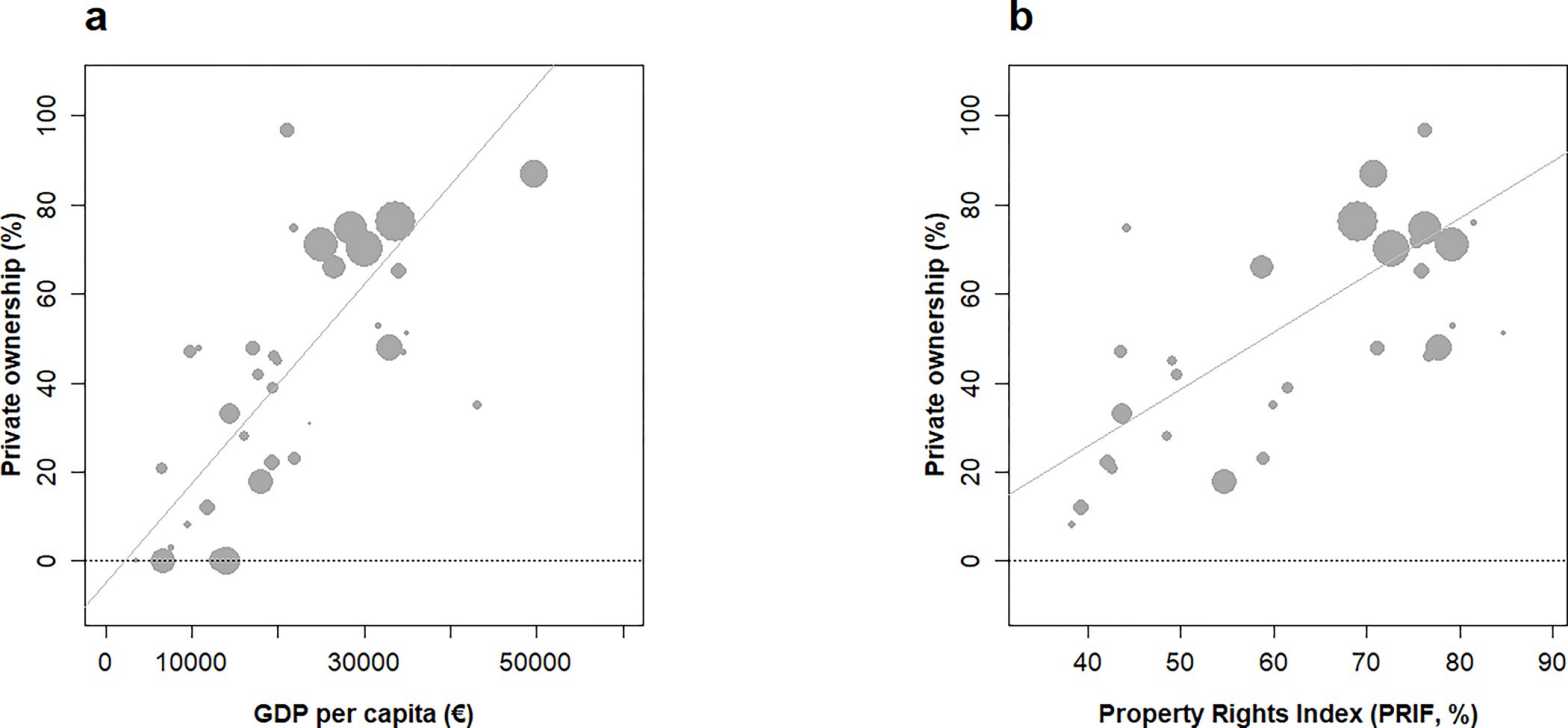
Relationships between private forest ownership fraction and economic richness (a) and freedom in decision making in forestry (b) across European countries under study. (a) economic development as measured by GDP.capita-1 (GDPc, euros) in 2013 across 39 countries (b) freedom in decision making as measured by the Property Rights Index in Forestry (PRIF, [42, 43]) in 2015 and available for 30 countries (see methods). Private ownership rate in 2010. Weighted correlations (forest area in 2015): (a) +0.8 (p < 10−8) and (b) +0.71 (p < 10−4).

**Fig 6:**
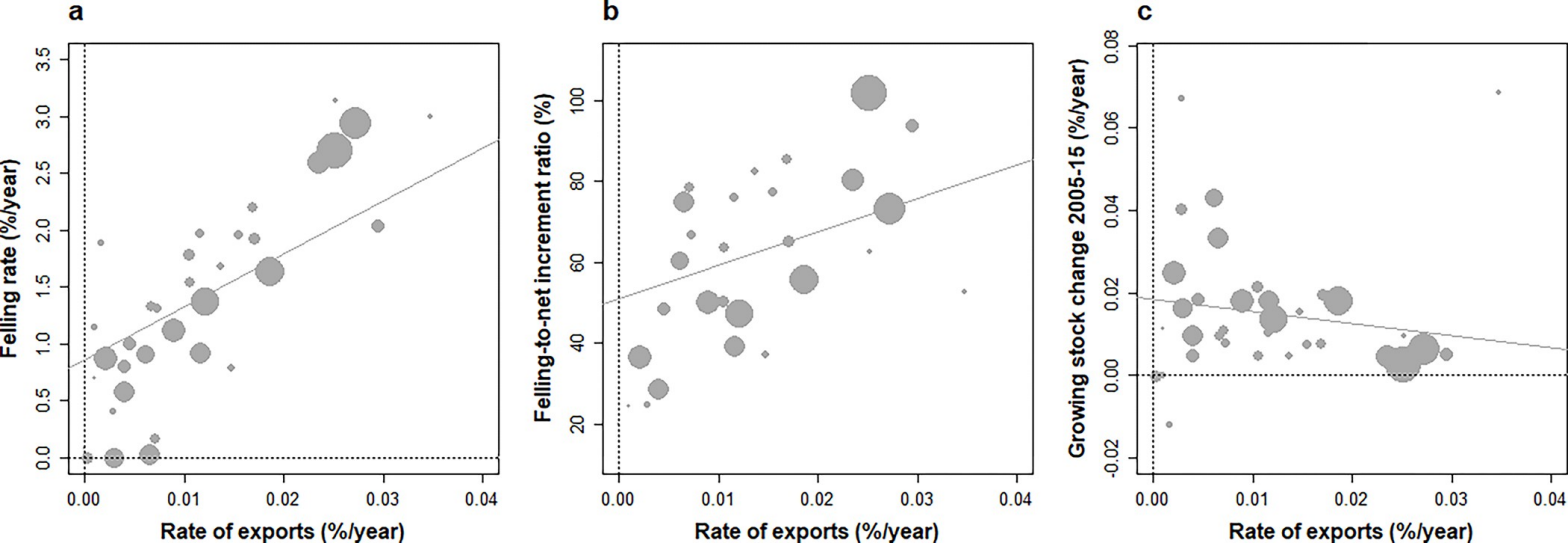
Relationships between the annual rates of wood exports and (a) felling rate, (b) felling-to-net increment ratio and (c) changes in the growing stock over 2005–15 across European countries under study. Both exports, felling and GS changes expressed as fractions (%) of GS volume in 2005. Weighted correlations (forest area in 2015): (a) +0.65 (p < 10–5), (b) + 0.49 (p < 0.01), (c) –0.34 (p < 0.05).
