# Supplementary figures and images for: Inflation of wood resources in European forests: The footprints of a big-bang
Source: PLoS One. 2021 Nov 24;16(11):e0259795. doi: 10.1371/journal.pone.0259795 (PMC8612577; doi:10.1371/journal.pone.0259795)

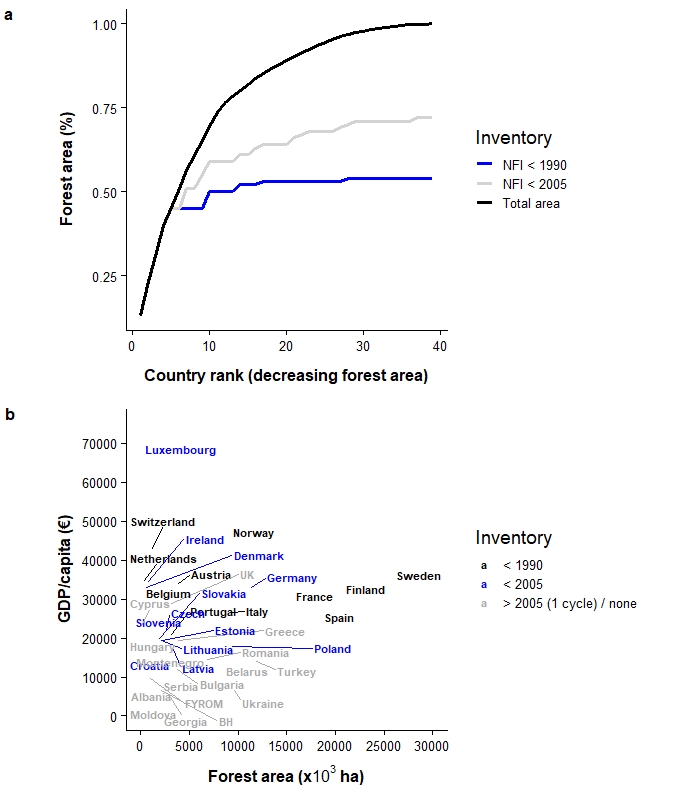

Supplement: S1 Fig — (a) Fraction of the European forest area covered by a national statistical forest inventory program in 1990 and 2005, (b) influence of countries’ richness and importance of forest area on their existence. (JPEG) [file pone.0259795.s001.jpeg]

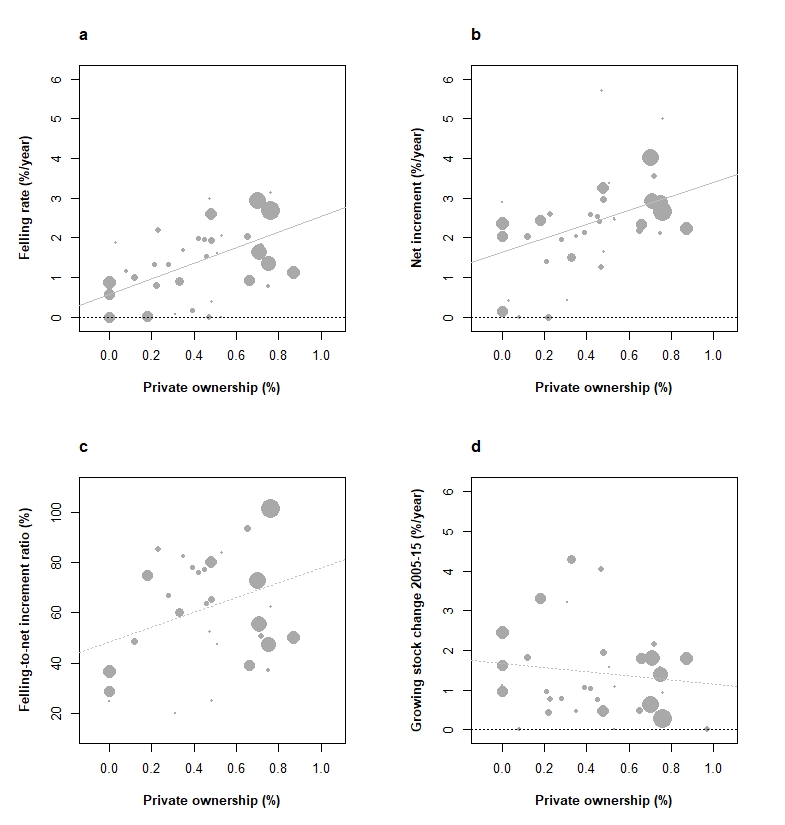

Supplement: S2 Fig — Relationships between private ownership rate (% forest area) and felling (a) and net increment (b) rates, felling-to-net-increment (FIR, c) and resulting changes in the growing stock over 2005–2015 (d) across 39 European countries under study. Private ownership rate in 2010. Albania was set aside (see Methods) Weighted correlations (forest area in 2015): (a) +0.60 (p < 10−4), (b) +0.55 (p < 10−3), (c) +0.36 (p < 0.05), -0.24 (p = 0.16). (JPEG) [file pone.0259795.s002.jpeg]

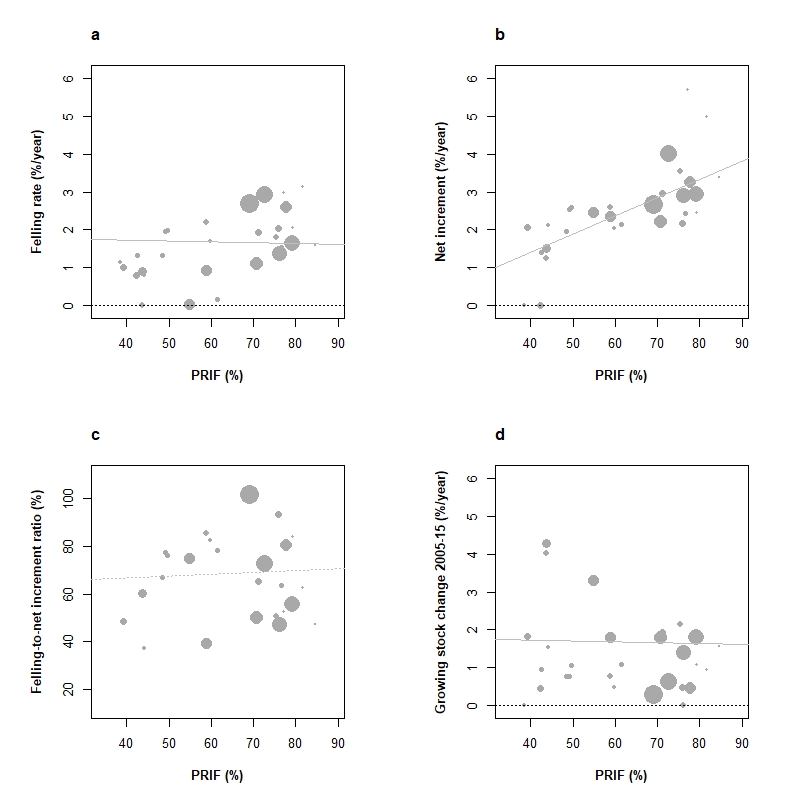

Supplement: S3 Fig — Relationships between the Property Rights Index for Forests (PRIF, scale ranging from 0 to 100) and felling and net increment rates (a, b), felling-to-net-increment (FIR, c) and resulting changes in the growing stock over 2005–2015 (d) across a subset of 30 out of 39 European countries under study. PRIF index in 2015, as defined in Nichiforel et al. 2018, and updated for 33 European countries in Nichiforel et al. 2020. Private ownership rate in 2010. PRIF in countries including Turkey, Ukraine, Belarus, Georgia, Montenegro, Albania, Moldova, Cyprus and Luxembourg was not documented. Weighted correlations (forest area in 2015): (a) +0.53 (p < 0.001), (b) +0.69 (p < 10−4), (c) +0.04 (NS), -0.37 (p = 0.04). (JPEG) [file pone.0259795.s003.jpeg]

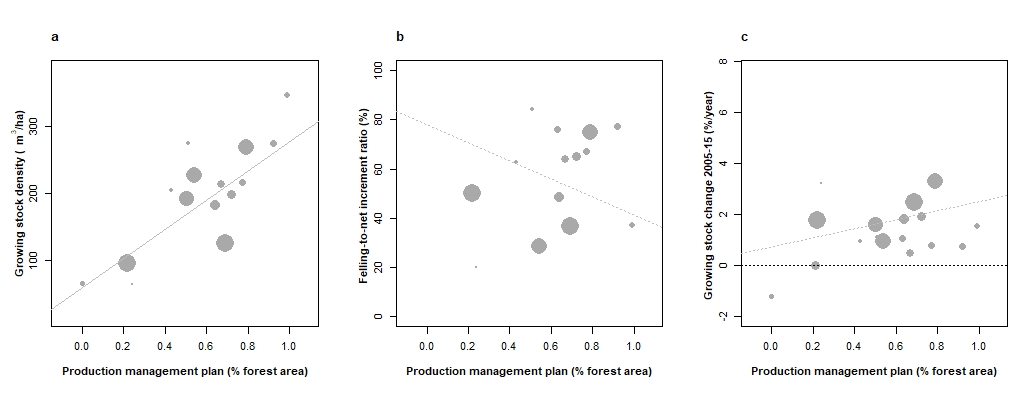

Supplement: S4 Fig — Relationship between forest area subjected to a production forest management plan (% forest area) and (a) growing stock per hectare (GSD, m3/ha), (b) felling-to-net increment ratio (%), and (c) growing stock change over 2005–2015 for 16 countries reporting on the indicator. FIR in 2010. GSD in 2015. Weighted correlations (forest area 2015) are (a) +0.71 (p < 0.01), (b) -0.17 (NS), (c) +0.43 (< 0.1). 12/16 countries with NFI programs covering the reporting period 2005–2015, 10/16 as EU members, 11/16 located in Eastern Europe: Ukraine, Belarus, Turkey, Bulgaria, Poland, Hungary, Croatia, Slovakia, Slovenia, Latvia and Estonia. (JPEG) [file pone.0259795.s004.jpeg]
