# Supplementary material for: Inflation of wood resources in European forests: The footprints of a big-bang
Source: PLoS One. 2021 Nov 24;16(11):e0259795. doi: 10.1371/journal.pone.0259795 (PMC8612577; doi:10.1371/journal.pone.0259795)
Supplement: S1 Table — See paper for country selection. a Data from Forest Europe 2015, b 1: 1990–2015, 2: 2005–2015, c C1 = 1st cycle of inventory, C2 = 2nd cycle of inventory. Reporting coverage by NFI statistics assumes that results of a previous NFI cycle were available prior to the starting year of a reporting period. d Information were obtained from Tomppo et al. 2010 (NFI–pathways for common reporting, Springer) and Vidal et al. 2016 (NFI–Assessment of wood availability and use, Springer) syntheses of European COST actions dedicated to national forest inventories. Links to additional resources are indicated when found. (DOCX) [file pone.0259795.s005.docx]

**S1 Table. Coverage by statistical national forest inventory (NFI) programmes of the *Forest Europe* reporting periods in the 40 European countries under study.** See paper for country selection. ^a^ Data from Forest Europe 2015, ^b^ 1: 1990-2015, 2: 2005-2015, ^c^ C1 = 1^st^ cycle of inventory, C2 = 2^nd^ cycle of inventory. Reporting coverage by NFI statistics assumes that results of a previous NFI cycle were available prior to the starting year of a reporting period. ^d^ Information were obtained from Tomppo et al. 2010 (NFI – pathways for common reporting, Springer) and Vidal et al. 2016 (NFI – Assessment of wood availability and use, Springer) syntheses of European COST actions dedicated to national forest inventories. Links to additional resources are indicated when found.

| **Rank** | **Country** | **Forest area 2015^a^** | **EU-28** | **NFI-covered reporting ^b^** | **Comment^c^** | **Additional source^d^** |
| --- | --- | --- | --- | --- | --- | --- |
| 1 | Sweden | 28,073 | 1 | 1,2 | - | [DOI](file:///C:\_DR\2_Recherche\7_Articles\r_SOEF\z_Article\dx.doi.org\10.14214\sf.1095) (Fridman et al. 2014) |
| 2 | Finland | 22,218 | 1 | 1,2 | - | GE |
| 3 | Spain | 18,418 | 1 | 1,2 | - | GE |
| 4 | France | 16,989 | 1 | 1,2 | - | GE |
| 5 | Norway | 12,112 | 0 | 1,2 | - | GE |
| 6 | Turkey | 11,943 | 0 | 0 | Planned | [DOI](file:///C:\_DR\2_Recherche\7_Articles\r_SOEF\z_Article\10.1007\978-94-017-0649-0) (Corona et al. 2003) |
| 7 | Germany | 11,419 | 1 | 2 | C1 1986 in W-Germany | GE |
| 8 | Ukraine | 9,657 | 0 | 0 | Planned | [REPORT](https://apd-ukraine.de/images/2018/APR/APD_APR_07-2017_Forest_Inventories_eng.pdf) (Ministerial) |
| 9 | Poland | 9,435 | 1 | 2 | C1 2005-09 | [REPORT](https://www.buligl.pl/web/en/national-forest-inventory) (NFI) |
| 10 | Italy | 9,297 | 1 | 1,2 | - | GE |
| 11 | Belarus | 8,633 | 0 | 0 | Inexistent | [website](http://www.fao.org/3/XII/0784-B1.htm) (FAO) |
| 12 | Romania | 6,861 | 1 | 0 | C1 2010 | [website](http://roifn.ro/site/about-nfi/) (NFI) |
| 13 | Greece | 3,903 | 1 | 0 | C1 1963-92, no C2 | GE |
| 14 | Austria | 3,869 | 1 | 1,2 | - | GE |
| 15 | Bulgaria | 3,823 | 1 | 0 | Envisioned | GE |
| 16 | Latvia | 3,356 | 1 | 2 | C1 2004-08, C2 2009-13 | GE |
| 17 | Portugal | 3,182 | 1 | 1,2 | - | GE |
| 18 | United-Kingdom | 3,144 | 1 | 0 | C1 2009-15 | GE, [website](https://www.forestresearch.gov.uk/tools-and-resources/national-forest-inventory/about-the-nfi/) (NFI) |
| 19 | Georgia | 2,822 | 0 | 0 | Envisioned | [REPORT](http://environment.cenn.org/app/uploads/2016/09/CENN-BROCHURE-reduced-ENG.pdf) (Ministerial) |
| 20 | Serbia | 2,720 | 0 | 0 | C1 2003, C2 ongoing 2015 | [REPORT](https://www.upravazasume.gov.rs/wp-content/uploads/2015/12/The-national-forest-inventory-of-the-Republic-of-Serbia.pdf) (Ministerial) |
| 21 | Czech Republic | 2,667 | 1 | 2 | C1 2000, C2 2006 | GE |
| 22 | Estonia | 2,232 | 1 | 2 | C1 2003, C2 2009 | GE |
| 23 | Lithuania | 2,180 | 1 | 2 | Continuous since 1998 | GE |
| 24 | B-Herzegovina | 2,115 | 0 | 0 | C1 in 2006, C2 in 2018 | GE |
| 25 | Hungary | 2,069 | 1 | 0 | C1 2010- | GE |
| 26 | Slovakia | 1,940 | 1 | 2 | C1 2004, C2015 | GE, [website](http://www.uhul.cz/what-we-do/national-forest-inventory) (Ministerial) |
| 27 | Croatia | 1,922 | 1 | 0 | C1 2005-09 | [REPORT](https://mzoe.gov.hr/UserDocsImages/KLIMA/SZKAIZOS/NFAP_Croatia.pdf) (Ministerial) |
| 28 | Switzerland | 1,254 | 0 | 1,2 | - | GE |
| 29 | Slovenia | 1,248 | 1 | 2 | C1 2000 | GE |
| 30 | FYROM | 987 | 0 | 0 | Planned | [website](http://www.fao.org/europe/news/detail-news/en/c/1100670/) (FAO) |
| 31 | Montenegro | 827 | 0 | 0 | C1 2010 | [REPORT](http://www.fao.org/3/a-az279e.pdf) (NFI) |
| 32 | Albania | 785 | 0 | 0 | Planned | [website](https://www.slu.se/en/ew-news/2016/10/slu-to-set-up-national-forest-inventory-in-albania/) (SLU) |
| 33 | Ireland | 754 | 1 | 2 | C1 2006, C2 2012 | [website](https://www.agriculture.gov.ie/nfi/) (NFI) |
| 34 | Belgium | 683 | 1 | 1,2 | - | GE, [website](http://iprfw.spw.wallonie.be/historique.php) (NFI) |
| 35 | Denmark | 612 | 1 | 2 | C1 2006, C2 2011 | GE |
| 36 | Moldova | 409 | 0 | 0 | Planned | [website](http://www.fao.org/europe/news/detail-news/en/c/1197689/) (FAO) |
| 37 | Netherlands | 376 | 1 | 1,2 | - | GE |
| 38 | Cyprus | 173 | 1 | 0 | Partial inventory | GE |
| 39 | Luxembourg | 87 | 1 | 2 | C1 1999 | [REPORT](https://statistiques.public.lu/fr/publications/thematique/territoire-environnement/forets-chiffres/IFL2interactif.pdf) (Ministerial) |
