# Supplementary material for: Inflation of wood resources in European forests: The footprints of a big-bang
Source: PLoS One. 2021 Nov 24;16(11):e0259795. doi: 10.1371/journal.pone.0259795 (PMC8612577; doi:10.1371/journal.pone.0259795)
Supplement: S2 Table — Significant correlations are highlighted in grey (trivial correlations associated to similar proxies or basic size effects, e. g. forest area and growing stock), in red (level of significance of correlation t-test <0.01) or green (level of significance <0.05). The test statistics of a Pearson correlation is given by t = r2/(1-r2)1/2 (n-2)1/2 where r is the correlation and n the number of sampling units (n = 39, see S1 Table), yielding: r = t / (t2 + n– 2)1/2. Associated correlations thresholds: |r| > 0.32 (p < 0.05) and |r| > 0.41 (p < 0.01). Indicators: A_country = country area (km2), EU28/NFI: binary variables indicating EU members and implementation of an NFI program, PopDens/RurPopDens = population density/rural population density (hab/km2), GDP = annual gross domestic product (€/capita), %Priv, %MgtPlan, %ProdMgtPlan = share of country forest area private/under a management plan/under a production management plan (%), A = forest area (km2), %AFor = country afforestation rate (%), GS = country forest growing stock (m3), GSD = country forest growing stock density (m3/ha), NI = forest net increment (m3), Fell = felling volume (m3), FIR = felling-to-net-increment ratio (%), %NI, %Fell = expressed as a percentage of total previous GS, ChgA/ChgGS = absolute changes in A/GS, %ChgA/%ChgGS = changes in A/GS expressed as a percentage of total previous GS, ChgGSha = change in GS expressed per unit of previous forest area. AccGS %/ha = acceleration in GS changes, computed as the difference between annual changes in GS over the two study periods 1990–2005 and 2005–15. Notations _YY/_YYYY refer to the calendar year (state variable) or calendar period (flux). (DOCX) [file pone.0259795.s006.docx]

**S2 Table. Correlation matrix of the indicators extracted and computed from *Forest Europe* data 2015**. Significant correlations are highlighted in grey (trivial correlations associated to similar proxies or basic size effects, e. g. forest area and growing stock), in red (level of significance of correlation *t*-test <0.01) or green (level of significance <0.05). The test statistics of a Pearson correlation is given by *t* = r²/(1-r²)^1/2^ (n-2)^1/2^ where r is the correlation and n the number of sampling units (n = 39, see supplementary table 1), yielding: r = *t* / (*t*² + n – 2)^1/2^. Associated correlations thresholds: |r| > 0.32 (p < 0.05) and |r| > 0.41 (p < 0.01).

|  | **A_Country** | **EU28** | **NFI** | **PopDens_13** | **RurPopDens_13** | **GDP_13** | **%Priv_10** | **%MgtPlan_10** | **%ProdMgtPlan_10** |
| --- | --- | --- | --- | --- | --- | --- | --- | --- | --- |
| **EU28** | -0,11 |  |  |  |  |  |  |  |  |
| **NFI** | 0,09 | 0,44 |  |  |  |  |  |  |  |
| **PopDens_13** | -0,11 | 0,24 | 0,31 |  |  |  |  |  |  |
| **RurPopDens_13** | -0,12 | -0,05 | -0,15 | 0,40 |  |  |  |  |  |
| **GDP_13** | -0,01 | 0,39 | **0,81** | 0,37 | -0,16 |  |  |  |  |
| **%Priv_10** | -0,03 | 0,49 | 0,75 | 0,12 | -0,15 | **0,59** |  |  |  |
| **%MgtPlan_10** | 0,09 | 0,01 | -0,59 | -0,12 | -0,05 | -0,47 | -0,57 |  |  |
| **%ProdMgtPlan_10** | 0,05 | 0,41 | -0,31 | -0,04 | 0,16 | -0,20 | -0,08 | 0,84 |  |
| **A_90** | 0,68 | 0,10 | **0,46** | -0,28 | -0,35 | 0,16 | 0,28 | -0,18 | -0,02 |
| **A_05** | 0,67 | 0,11 | **0,46** | -0,27 | -0,34 | 0,16 | 0,28 | -0,20 | 0,00 |
| **A_15** | 0,69 | 0,10 | **0,46** | -0,27 | -0,34 | 0,15 | 0,28 | -0,20 | 0,01 |
| **%AFor_15** | -0,11 | -0,01 | 0,23 | **-0,54** | -0,33 | -0,07 | 0,26 | -0,12 | 0,06 |
| **ChgA_9005** | 0,28 | 0,10 | 0,25 | -0,05 | -0,10 | -0,01 | 0,11 | -0,27 | 0,27 |
| **%ChgA_9005** | 0,00 | 0,12 | -0,05 | -0,05 | 0,01 | 0,04 | -0,07 | -0,12 | 0,34 |
| **ChgA_0515** | 0,54 | -0,03 | 0,07 | -0,04 | -0,03 | -0,09 | -0,02 | -0,09 | 0,21 |
| **%ChgA_0515** | 0,06 | -0,25 | -0,26 | -0,08 | -0,04 | -0,20 | -0,08 | 0,02 | 0,19 |
| **GS_90** | 0,73 | 0,14 | 0,35 | -0,17 | -0,12 | 0,12 | 0,15 | 0,08 | 0,26 |
| **GS_05** | 0,75 | 0,14 | 0,36 | -0,13 | -0,09 | 0,10 | 0,12 | 0,08 | 0,25 |
| **GS_15** | 0,77 | 0,15 | 0,38 | -0,13 | -0,06 | 0,08 | 0,15 | 0,05 | 0,21 |
| **GSD_05** | -0,09 | 0,20 | 0,18 | **0,36** | 0,24 | 0,32 | 0,01 | 0,46 | **0,80** |
| **GSD_15** | -0,09 | 0,27 | 0,20 | **0,35** | 0,29 | 0,28 | 0,10 | 0,46 | **0,81** |
| **NI_05** | 0,65 | 0,24 | 0,45 | -0,10 | -0,10 | 0,18 | 0,28 | -0,09 | 0,10 |
| **%NI_05** | 0,06 | 0,36 | 0,43 | 0,19 | 0,10 | 0,17 | **0,55** | -0,30 | -0,14 |
| **NI_10** | 0,67 | 0,24 | 0,45 | -0,09 | -0,06 | 0,14 | 0,23 | -0,06 | 0,26 |
| **%NI_10** | 0,16 | 0,48 | 0,56 | 0,22 | -0,07 | **0,45** | **0,51** | -0,08 | **0,36** |
| **Fell_10** | 0,48 | 0,26 | 0,50 | -0,07 | -0,14 | **0,36** | 0,34 | -0,06 | 0,07 |
| **%Fell_10** | 0,00 | 0,38 | 0,60 | 0,12 | -0,12 | 0,29 | **0,40** | -0,16 | -0,07 |
| **FIR_10** | -0,16 | -0,20 | -0,08 | -0,03 | 0,07 | -0,15 | -0,22 | -0,27 | **-0,54** |
| **ChgGS_9015** | 0,75 | 0,08 | 0,22 | -0,12 | -0,02 | -0,02 | 0,04 | 0,05 | 0,21 |
| **%ChgGS_9015** | 0,17 | 0,22 | 0,03 | 0,17 | -0,02 | 0,04 | 0,24 | 0,05 | **0,36** |
| **ChgGS_9005** | 0,69 | 0,05 | 0,25 | -0,09 | -0,09 | 0,05 | 0,04 | 0,05 | 0,23 |
| **%ChgGS_9005** | 0,09 | 0,21 | 0,08 | 0,20 | -0,15 | 0,18 | 0,18 | 0,10 | **0,43** |
| **ChgGSha_9005** | 0,01 | 0,29 | 0,09 | 0,37 | 0,04 | 0,28 | 0,14 | **0,36** | **0,63** |
| **ChgGS_0515** | 0,61 | 0,08 | 0,05 | -0,11 | 0,07 | -0,09 | -0,06 | 0,11 | 0,23 |
| **%ChgGS_0515** | 0,05 | 0,00 | -0,22 | -0,10 | -0,03 | -0,09 | 0,06 | 0,16 | 0,25 |
| **ChgGSha_0515** | 0,07 | 0,10 | -0,21 | 0,02 | 0,11 | -0,14 | 0,03 | **0,38** | **0,63** |
| **AccGS%_9015** | 0,13 | -0,12 | -0,29 | -0,16 | 0,20 | -0,26 | -0,23 | 0,12 | -0,12 |
| **AccGSha_9015** | 0,16 | -0,07 | -0,25 | -0,17 | 0,14 | -0,35 | -0,19 | 0,11 | -0,02 |

Indicators: A_country = country area (km²), EU28/NFI: binary variables indicating EU members and implementation of an NFI program, PopDens/RurPopDens = population density/rural population density (hab/km²), GDP = annual gross domestic product (€/capita), %Priv, %MgtPlan, %ProdMgtPlan = share of country forest area private/under a management plan/under a production management plan (%), A = forest area (km²), %AFor = country afforestation rate (%), GS = country forest growing stock (m^3^), GSD = country forest growing stock density (m^3^/ha), NI = forest net increment (m^3^), Fell = felling volume (m^3^), FIR = felling-to-net-increment ratio (%), %NI, %Fell = expressed as a percentage of total previous GS, ChgA/ChgGS = absolute changes in A/GS, %ChgA/%ChgGS = changes in A/GS expressed as a percentage of total previous GS, ChgGSha = change in GS expressed per unit of previous forest area. AccGS %/ha = acceleration in GS changes, computed as the difference between annual changes in GS over the two study periods 1990-2005 and 2005-15. Notations _YY/_YYYY refer to the calendar year (state variable) or calendar period (flux).

|  | **A_90** | **A_05** | **A_15** | **%AFor_15** | **ChgA_9005** | **%ChgA_9005** | **ChgA_0515** | **%ChgA_0515** | **GS_90** | **GS_05** | **GS_15** | **GSD_05** | **GSD_15** |
| --- | --- | --- | --- | --- | --- | --- | --- | --- | --- | --- | --- | --- | --- |
| **A_05** | 1,00 |  |  |  |  |  |  |  |  |  |  |  |  |
| **A_15** | 0,99 | 1,00 |  |  |  |  |  |  |  |  |  |  |  |
| **%AFor_15** | 0,40 | 0,39 | 0,38 |  |  |  |  |  |  |  |  |  |  |
| **ChgA_9005** | 0,39 | 0,47 | 0,50 | 0,07 |  |  |  |  |  |  |  |  |  |
| **%ChgA_9005** | -0,05 | -0,01 | 0,01 | -0,33 | 0,47 |  |  |  |  |  |  |  |  |
| **ChgA_0515** | 0,31 | 0,38 | 0,42 | -0,10 | 0,81 | **0,34** |  |  |  |  |  |  |  |
| **%ChgA_0515** | -0,12 | -0,10 | -0,08 | -0,14 | 0,21 | **0,37** | 0,42 |  |  |  |  |  |  |
| **GS_90** | 0,84 | 0,83 | 0,82 | 0,21 | 0,21 | -0,10 | 0,27 | -0,05 |  |  |  |  |  |
| **GS_05** | 0,84 | 0,83 | 0,83 | 0,17 | 0,26 | -0,13 | 0,28 | -0,16 | 0,99 |  |  |  |  |
| **GS_15** | 0,83 | 0,82 | 0,82 | 0,15 | 0,28 | -0,14 | 0,33 | -0,15 | 0,99 | 0,99 |  |  |  |
| **GSD_05** | -0,23 | -0,25 | -0,25 | -0,25 | -0,28 | -0,30 | -0,22 | -0,23 | 0,09 | 0,12 | 0,09 |  |  |
| **GSD_15** | -0,25 | -0,27 | -0,28 | -0,24 | -0,32 | -0,30 | -0,23 | -0,23 | 0,09 | 0,11 | 0,12 | 0,98 |  |
| **NI_05** | 0,81 | 0,79 | 0,79 | 0,21 | 0,24 | -0,03 | 0,24 | -0,15 | 0,94 | 0,93 | 0,92 | -0,01 | -0,03 |
| **%NI_05** | 0,10 | 0,10 | 0,09 | -0,03 | -0,01 | -0,14 | -0,05 | -0,15 | 0,01 | 0,01 | 0,21 | -0,15 | 0,11 |
| **NI_10** | 0,82 | 0,81 | 0,80 | 0,20 | 0,25 | -0,10 | 0,26 | -0,15 | 0,94 | 0,94 | 0,93 | 0,01 | 0,00 |
| **%NI_10** | 0,17 | 0,18 | 0,18 | -0,32 | 0,09 | 0,37 | 0,06 | 0,18 | 0,26 | 0,17 | 0,16 | 0,20 | 0,23 |
| **Fell_10** | 0,79 | 0,77 | 0,76 | 0,32 | 0,11 | -0,17 | 0,05 | -0,22 | 0,87 | 0,86 | 0,81 | 0,10 | 0,06 |
| **%Fell_10** | 0,25 | 0,24 | 0,22 | -0,01 | -0,03 | 0,12 | -0,15 | -0,13 | 0,27 | 0,20 | 0,15 | 0,09 | 0,06 |
| **FIR_10** | -0,05 | -0,06 | -0,07 | 0,12 | -0,13 | -0,21 | -0,18 | -0,26 | -0,09 | -0,08 | -0,09 | -0,10 | -0,16 |
| **ChgGS_9015** | 0,67 | 0,68 | 0,69 | 0,00 | **0,41** | 0,11 | **0,47** | 0,11 | **0,82** | **0,86** | **0,91** | 0,02 | 0,10 |
| **%ChgGS_9015** | 0,04 | 0,07 | 0,09 | -0,30 | **0,36** | **0,39** | **0,32** | **0,42** | -0,01 | 0,05 | 0,11 | -0,01 | 0,09 |
| **ChgGS_9005** | 0,70 | 0,71 | 0,71 | 0,06 | **0,41** | 0,15 | 0,31 | -0,01 | **0,84** | **0,89** | **0,88** | 0,08 | 0,06 |
| **%ChgGS_9005** | 0,06 | 0,09 | 0,10 | -0,30 | **0,36** | **0,39** | 0,20 | **0,33** | 0,01 | 0,09 | 0,06 | 0,20 | 0,15 |
| **ChgGSha_9005** | -0,16 | -0,15 | -0,15 | -0,37 | -0,02 | 0,02 | -0,08 | 0,06 | 0,02 | 0,08 | 0,04 | **0,65** | **0,63** |
| **ChgGS_0515** | 0,43 | 0,45 | 0,46 | -0,09 | 0,31 | 0,00 | **0,54** | 0,11 | **0,56** | **0,57** | **0,67** | 0,00 | 0,12 |
| **%ChgGS_0515** | -0,11 | -0,10 | -0,09 | -0,12 | 0,06 | **0,46** | 0,23 | **0,69** | 0,06 | -0,11 | -0,05 | -0,24 | -0,08 |
| **ChgGSha_0515** | -0,14 | -0,14 | -0,13 | -0,19 | -0,07 | 0,22 | 0,17 | **0,54** | 0,12 | -0,01 | 0,07 | 0,10 | 0,28 |
| **AccGS%_9015** | -0,01 | -0,03 | -0,02 | 0,04 | -0,14 | -0,06 | 0,18 | 0,14 | 0,07 | 0,01 | 0,08 | -0,22 | -0,11 |
| **AccGSha_9015** | 0,07 | 0,07 | 0,09 | 0,00 | 0,01 | 0,04 | 0,28 | 0,27 | 0,11 | 0,06 | 0,16 | -0,25 | -0,10 |

**S2 Table (continued).**

|  | **NI_05** | **%NI_05** | **NI_10** | **%NI_10** | **Fell_10** | **%Fell_10** | **FIR_10** | **ChgGS_9015** | **%ChgGS_9015** |
| --- | --- | --- | --- | --- | --- | --- | --- | --- | --- |
| **%NI_05** | 0,22 |  |  |  |  |  |  |  |  |
| **NI_10** | 1,00 | 0,22 |  |  |  |  |  |  |  |
| **%NI_10** | 0,39 | 0,94 | 0,28 |  |  |  |  |  |  |
| **Fell_10** | 0,95 | 0,17 | 0,89 | 0,29 |  |  |  |  |  |
| **%Fell_10** | **0,46** | **0,57** | 0,31 | **0,66** | 0,51 |  |  |  |  |
| **FIR_10** | -0,08 | -0,34 | -0,07 | -0,31 | 0,01 | 0,24 |  |  |  |
| **ChgGS_9015** | 0,76 | 0,11 | 0,76 | 0,20 | 0,52 | -0,05 | -0,21 |  |  |
| **%ChgGS_9015** | -0,11 | 0,22 | -0,06 | **0,40** | -0,17 | -0,15 | **-0,67** | 0,37 |  |
| **ChgGS_9005** | 0,78 | -0,07 | 0,79 | 0,25 | 0,68 | 0,11 | -0,18 | 0,89 | 0,28 |
| **%ChgGS_9005** | -0,04 | -0,09 | -0,02 | **0,53** | -0,04 | 0,08 | **-0,56** | 0,25 | 0,87 |
| **ChgGSha_9005** | -0,06 | -0,09 | -0,04 | **0,52** | 0,00 | 0,10 | **-0,44** | 0,16 | 0,60 |
| **ChgGS_0515** | 0,43 | 0,03 | 0,48 | 0,05 | 0,17 | -0,22 | -0,16 | 0,84 | 0,37 |
| **%ChgGS_0515** | -0,18 | -0,19 | -0,15 | 0,24 | -0,24 | -0,16 | **-0,43** | 0,37 | 0,70 |
| **ChgGSha_0515** | -0,17 | -0,10 | -0,10 | 0,23 | -0,24 | -0,16 | **-0,36** | 0,41 | 0,57 |
| **AccGS%_9015** | -0,05 | **-0,36** | 0,00 | **-0,39** | -0,14 | -0,39 | 0,04 | 0,10 | -0,12 |
| **AccGSha_9015** | -0,04 | -0,22 | 0,03 | -0,27 | -0,17 | -0,29 | 0,02 | 0,25 | 0,06 |

**S2 Table (continued).**

|  | **ChgGS_9005** | **%ChgGS_9005** | **ChgGSha_9005** | **ChgGS_0515** | **%ChgGS_0515** | **ChgGSha_0515** | **AccGS%_9015** |
| --- | --- | --- | --- | --- | --- | --- | --- |
| **%ChgGS_9005** | 0,37 |  |  |  |  |  |  |
| **ChgGSha_9005** | 0,31 | 0,81 |  |  |  |  |  |
| **ChgGS_0515** | 0,49 | 0,07 | -0,03 |  |  |  |  |
| **%ChgGS_0515** | 0,06 | 0,26 | 0,05 | 0,34 |  |  |  |
| **ChgGSha_0515** | 0,06 | 0,20 | 0,24 | 0,55 | 0,86 |  |  |
| **AccGS%_9015** | -0,23 | **-0,59** | **-0,60** | **0,48** | **0,62** | **0,53** |  |
| **AccGSha_9015** | -0,15 | **-0,40** | **-0,52** | **0,65** | **0,69** | **0,70** | 0,91 |

**S2 Table (end).**
