# Supplementary material for: Inflation of wood resources in European forests: The footprints of a big-bang
Source: PLoS One. 2021 Nov 24;16(11):e0259795. doi: 10.1371/journal.pone.0259795 (PMC8612577; doi:10.1371/journal.pone.0259795)
Supplement: S3 Table — Aboveground forest carbon stocks stem from Paul T., Kimberley M.O., Beets P. N. Natural forests in New Zealand–a large terrestrial carbon pool in a national state of equilibrium. Forest Ecosystems (2021): 8:34. These forests are broadleaved with a Fagus genus as a dominant tree species, thus a wood specific gravity of 600 kg/m3 was used for biomass to volume conversion, using Fagus sylvatica data from Kerfriden B. et al., 2021, Plant Ecology 222:289–303, DOI: 10.1007/s11258-020-01106-0. The ratio of total aerial tree volume to total stem volume has been estimated at 1.27 in broadleaved semi-natural forests of France including 49 tree species and it was used for stem volume conversion using Saint-André L. et al., 2010, in Loustau D. (ed) Forests, carbon cycle and climate change, Quae ed, Paris, p79, Table 4.1. Last, conversion of total C stock (including roots, litter and deadwood) to aboveground C stock in Paul et al. arise from the constant 0.6 fraction established from this reference, S3 Table. (DOCX) [file pone.0259795.s007.docx]

**S3 Table. Conversion of aboveground forest carbon stocks encountered in natural forests of New-Zealand as aboveground forest GS in stem volume.**

Aboveground forest carbon stocks stem from Paul T., Kimberley M.O., Beets P. N. Natural forests in New Zealand – a large terrestrial carbon pool in a national state of equilibrium. *Forest Ecosystems* (2021): 8:34. These forests are broadleaved with a *Fagus* genus as a dominant tree species, thus a wood specific gravity of 600 kg/m^3^ was used for biomass to volume conversion, using *Fagus sylvatica* data from Kerfriden B. et al., 2021, *Plant Ecology* 222:289–303, DOI: [10.1007/s11258-020-01106-0](https://doi.org/10.1007/s11258-020-01106-0). The ratio of total aerial tree volume to total stem volume has been estimated at 1.27 in broadleaved semi-natural forests of France including 49 tree species and it was used for stem volume conversion using [Saint-André L. et al., 2010](https://www.quae.com/produit/135/9782759209897/forests-carbon-cycle-and-climate-change), *in* Loustau D. (ed) Forests, carbon cycle and climate change, *Quae* ed, Paris, p79, Table 4.1. Last, conversion of total C stock (including roots, litter and deadwood) to aboveground C stock in Paul et al. arise from the constant 0.6 fraction established from this reference, Table 3.

| **Indicator** | **Unit** | **Average**  ***Tall natural forests of New-Zealand (Table 3)*** | **Minimum**  ***Tall Beech forest of New-Zealand (Table 5)*** | **Maximum**  ***Tall Broadleaved podocarp forest (Table 5)*** | **Reference** |
| --- | --- | --- | --- | --- | --- |
| **Total carbon mass** | T carbon/ha |  | 191 | 316 | Paul et al. 2021, Table 5 |
| **Aboveground C mass (AGB)** | T carbon/ha | 149 | 115 | 190 | Paul et al. 2021, Table 3 / 0.6 fraction for converting Total C to AGB |
| **Aboveground biomass** | T biomass/ha | 298 | 230 | 380 | C fraction 0.5 |
| **Aboveground tree aerial volume** | m^3^/ha | 497 | 383 | 633 | Kerfriden et al. 2021 |
| **Aboveground stem aerial volume** | m^3^/ha | **391** | **302** | **499** | Saint-André et al. 2010, Table 4.1 |
